# Exosome‐Based Therapeutics: A Natural Solution to Overcoming the Blood–Brain Barrier in Neurodegenerative Diseases

**DOI:** 10.1002/mco2.70386

**Published:** 2025-09-12

**Authors:** Min Sun, Feng Qin, Qian Bu, Yue Zhao, Xiaofeng Yang, Dingwen Zhang, Xiaobo Cen

**Affiliations:** ^1^ Mental Health Center and National Chengdu Center for Safety Evaluation of Drugs State Key Laboratory of Biotherapy and Collaborative Innovation Center for Biotherapy West China Hospital, Sichuan University Chengdu China

**Keywords:** blood–brain barrier, drug delivery systems, exosomes, neurodegeneration therapy, neurodegenerative diseases

## Abstract

Neurodegenerative diseases present significant therapeutic challenges, primarily due to the restrictive nature of the blood–brain barrier (BBB), which limits drug delivery to the brain. While the BBB is crucial for protecting the brain from harmful substances, it also hinders the effectiveness of treatments for neurodegenerative diseases. Consequently, there is an urgent need for innovative drug delivery systems capable of bypassing the BBB to improve therapeutic outcomes. Exosomes, as endogenous nanoscale carriers, offer substantial promise for brain‐targeted drug delivery. Their unique characteristics, including the ability to cross biological barriers, high biocompatibility, intrinsic targeting capacity, natural intracellular transport mechanisms, and robust stability, render them highly promising candidates for drug delivery in the treatment of neurodegenerative disorders. This review delves into various engineering strategies for exosome‐mediated targeted drug delivery and provides an in‐depth analysis of the structural and functional properties of the BBB under normal and pathological conditions. We emphasize the potential of exosomes as drug delivery vehicles for the central nervous system, particularly in addressing neurodegenerative disorders. Furthermore, we address the key obstacles to the clinical application of exosome‐based therapies and propose future research directions aimed at optimizing these methods to develop more effective treatment strategies.

## Graphical abstract

Therapies for neurodegenerative diseases (NDs) are constrained by the blood–brain barrier (BBB), which limits drug delivery. Exosomes, with their ability to cross BBB and deliver drugs efficiently, emerge as a promising platform for central nervous system‐targeted therapies.

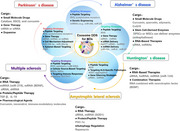

## 1 Introduction

The treatment of neurodegenerative diseases (NDs) remains a significant challenge, particularly in the context of delivering drugs to the brain. One of the primary barriers to effective therapy is the blood–brain barrier (BBB), a complex, selectively permeable structure that poses substantial challenges to the treatment of central nervous system (CNS) disorders. While the BBB serves a crucial protective role by restricting the entry of potentially harmful substances into the brain, it also significantly limits the ability of therapeutic agents to penetrate and exert their intended effects. Structurally, the BBB is composed of brain capillary endothelial cells (ECs), the basement membrane, and astrocytic end‐feet. One of the key features limiting drug permeability is the presence of tightly regulated intercellular tight junctions (TJs) between ECs, which serve as a physical barrier to paracellular transport. These TJs are not static but are functionally regulated by various signaling pathways and pathological stimuli, thereby influencing drug penetration into the brain [1]. In addition to the physical barrier formed by TJs, the BBB possesses active biochemical barriers mediated by efflux transporters such as multidrug resistance protein 1 (MDR1, also known as P‐glycoprotein [P‐gp]) expressed at the apical membrane of ECs. MDR1 actively pumps a broad range of low‐molecular‐weight, hydrophobic compounds back into the bloodstream, thereby limiting their passive diffusion into the brain parenchyma. Without MDR1 and related efflux transporters, many compounds could easily cross into the brain via passive diffusion, leading to potential neurotoxicity [2]. As a result, most therapeutic agents developed for the treatment of NDs and brain tumors are unable to efficiently access the brain via systemic circulation, which significantly restricts their clinical application. There is a critical demand for novel drug delivery systems (DDSs) capable of traversing the BBB to improve the efficacy of these therapies [3, 4, 5].

Historically, design of synthetic nanocarrier has focused on improving therapeutic efficacy and optimizing pharmacokinetics and pharmacodynamics, and minimizing systemic toxicity and side effects [6, 7]. To achieve these objectives, researchers have developed novel DDSs, including functionalized, stimuli‐responsive, and targeted lipid‐ or polymer‐based nanoparticles. These platforms offer notable advantages, such as extended circulation time, improved biodistribution, enhanced cellular interactions, and controlled drug loading and release. However, despite these advancements, synthetic nanocarriers still face significant challenges. These include insufficient targeting specificity, potential cytotoxicity, elevated immunogenicity, and limited therapeutic efficacy [8]. Among these systems, lipid nanoparticles (LNPs) have gained significant attention for their ability to encapsulate, protect, and deliver therapeutic agents to targeted sites.

To improve LNP stability and circulation time, polyethylene glycol (PEG) conjugation is commonly employed. However, repeated administration of PEGylated carriers often results in complications, including the phenomenon of accelerated blood clearance, decreased targeting accuracy, increased immune response, and reduced cellular uptake, all of which collectively hinder clinical translation [9, 10, 11, 12]. Other commonly used synthetic carriers, such as polymeric and inorganic nanoparticles, also present inherent limitations. These include cytotoxicity, rapid clearance by the mononuclear phagocyte system (MPS), limited ability to cross the BBB, and concerns regarding immunogenicity and long‐term safety, which further limit their clinical applicability [13, 14]. Liposomes, one of the earliest lipid‐based carriers, remain important due to their structural flexibility and accessible raw materials. Encapsulating drugs within liposomal membranes greatly enhances pharmacokinetics and protects drugs from degradation, inactivation, or dilution in the bloodstream [15]. Integrating LNP technology with other nanocarrier platforms has further improved delivery efficiency, tissue specificity, retention, and bioavailability, while mitigating several limitations of traditional carriers. Nevertheless, the clinical use of lipid‐based nanocarriers remains limited by poor bioavailability, potential toxicity, rapid clearance, and immune activation. Future research should prioritize improving their biocompatibility, safety, and targeting precision to facilitate clinical translation in precision medicine.

Since their discovery, exosomes have been increasingly recognized for their structural resemblance to traditional liposomes. They can be considered biologically derived, more complex analogs of natural liposomes. Despite shared features, exosomes offer distinct advantages that make them superior candidates for drug delivery. A major advantage lies in their lipid membrane, which contains a high proportion of lipids that prevent the formation of lamellar structures. This unique composition induces membrane curvature, a property shown to significantly enhance the loading and release efficiency of therapeutic agents [3, 4]. Initially dismissed as cellular waste and dubbed “platelet dust” by Peter Wolf in 1967 [16], extracellular vesicles (EVs) are now acknowledged as efficient mediators of intercellular signaling and targeted drug delivery. EVs are categorized into exosomes, microvesicles, and apoptotic bodies, depending on their size and the release mechanisms involved. Among these, EVs ranging in size from 30 to 150 nm and formed through endocytosis present distinct advantages over synthetic DDSs, particularly in modulating intricate physiological and pathological processes [17].

Exosomes can be engineered to deliver targeted molecules, thereby reducing systemic toxicity and enhancing drug safety and stability [18, 19, 20]. As naturally occurring vesicles, exosomes exhibit high biocompatibility, low immunogenicity, and a remarkable ability to cross biological barriers, including the BBB [20, 21, 22, 23]. Their potential has garnered increasing attention, particularly in the context of ND treatment [24].

NDs encompass a range of debilitating CNS disorders, such as Parkinson's disease (PD), Alzheimer's disease (AD), Huntington's disease (HD), amyotrophic lateral sclerosis (ALS), and multiple sclerosis (MS). These conditions, marked by the gradual loss of neurons, pose a significant risk to human health. Although some insight into the pathogenesis of these diseases has been gained, their exact mechanisms remain unclear, and current treatment options are limited. For instance, AD affects over 50 million people worldwide, while the prevalence of PD is projected to double by 2050 [25, 26]. HD affects approximately 30,000 people in the United States. With global aging accelerating, the prevalence of NDs is expected to increase to 12 million within the next 30 years, further exacerbating public health concerns [27]. The estimated prevalence of ALS ranges from four to eight cases per 100,000 individuals, with notable differences observed among various ethnic groups [28]. Meanwhile, the global prevalence of MS is increasing, particularly in Europe [29].

Given these challenges, exosomes have emerged as a promising therapeutic platform. Their ability to transport exogenous substances, maintain homeostasis, penetrate target cells, and release therapeutic agents makes them ideal drug carriers [30, 31, 32]. Exosomes' capability to traverse the BBB underscores their potential as a breakthrough therapy for CNS disorders. Furthermore, their lipid bilayer protects against immune surveillance and enzymatic degradation [33]. Recent studies highlights the pivotal role of exosomes in the progression, diagnosis, and treatment of neurological disorders [34, 35]. Moreover, their potential as drug delivery vehicles for brain tumors and other neurological diseases is actively being investigated [36, 37, 38, 39]. Despite these advancements, a comprehensive review addressing the therapeutic potential and challenges associated with exosomes in the treatment of NDs.

This review will examine the biochemical characteristics of exosomes and their ability to traverse the BBB, assessing their therapeutic potential in PD, AD, HD, ALS, and MS. It will address the current challenges in the field and propose future research directions.

## 2 The Biochemical Characteristics of Brain‐Targeted Exosomes

The study of exosomes dates back to the 1980s. In 1946, Chargaff and West [40] demonstrated that the removal of the plasma fraction after high‐speed centrifugation inhibited coagulation in human plasma. Subsequently, Wolf [16] identified that these coagulation inhibitors were 20–50 nm vesicles derived from platelets. However, it was not until 1983 that Johnstone's team first observed, using electron microscopy, membrane‐bound vesicles ranging from 30 to 100 nm in diameter, released from multivesicular bodies (MVBs) into the extracellular space, and named them “exosomes” [41]. Initially, these vesicles were regarded merely as waste products of cellular excretion, which limited their scientific attention. With the advancement of ultracentrifugation techniques and omics technologies in the 21st century, research on exosomes saw remarkable growth. Recent studies have confirmed that exosomes are involved in numerous crucial physiological and pathological processes, including the propagation of NDs and the modulation of tumor microenvironments. As a result, exosomes have emerged as a promising target for both disease diagnosis and therapeutic interventions. This section will explore the biogenesis, molecular composition, biological functions, separation and purification techniques, characterization methods, unique advantages in brain delivery, and strategies for drug loading of exosomes, with the aim of providing a comprehensive reference for an in‐depth understanding of the physicochemical properties of exosomes and their potential for clinical translation.

### 2.1 Exosomes

#### 2.1.1 The Biogenesis of Exosomes

**FIGURE 1:**
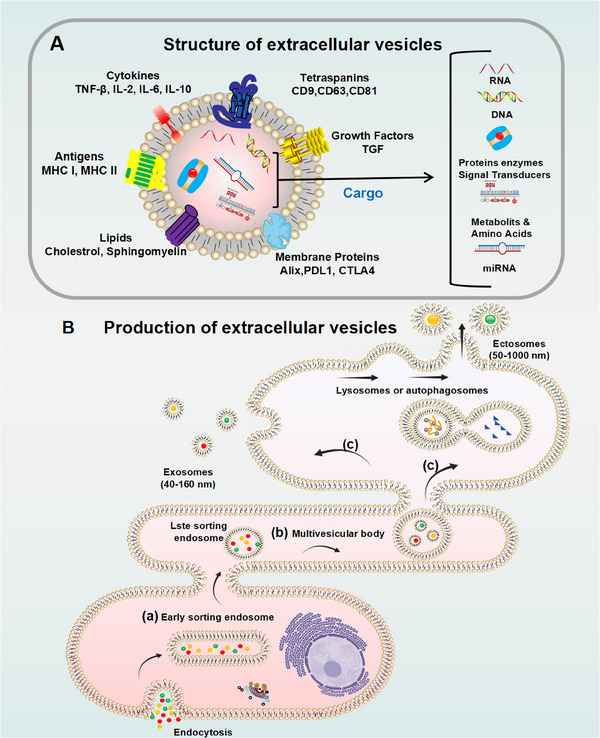
Synthesis and composition of exosomes. EVs, encapsulated by a lipid bilayer, are generally divided into two primary types: ectosomes and exosomes. Ectosomes, with sizes ranging from 50 nm to 1 µm, are produced through outward budding of the plasma membrane. In contrast, exosomes, typically measuring between 40 and 160 nm, are generated from endosomes through a three‐step process. (a) In the first step, plasma membrane endocytosis results in the formation of ESEs, where membrane components and extracellular substances are internalized. (b) In the second step, ESEs mature into LSEs, which involves the inward budding of the endosomal membrane, leading to the formation of MVBs containing numerous ILVs. (c) In the final step, MVBs either fuse with lysosomes or autophagosomes for degradation, or they merge with the plasma membrane, releasing ILVs as exosomes. The molecular composition of exosomes, which typically includes nucleic acids, proteins, membrane proteins (such as tetraspanins), antigens, cytokines, growth factors, and lipids, is determined by their cellular origin and the activation state of the cell. EVs, extracellular vesicles; ESEs, early sorting endosomes; CTLA4, cytotoxic T‐lymphocyte‐associated protein 4; IL, interleukin; ILVs, intraluminal vesicles; LSEs, late sorting endosomes; MHC, major histocompatibility complex; MVB, multivesicular body; MVBs, multivesicular bodies; PDL1, programmed death‐ligand 1; TGF, transforming growth factor.

Exosomes are key mediators of intercellular communication. Initially, they were mistakenly considered to be simple “waste disposals,” responsible for packaging cellular debris into vesicles and releasing it into the extracellular space via plasma membrane fusion [42, 43]. However, it is now well established that exosomes are integral to intercellular exchange, contribute to disease propagation, and hold significant therapeutic promise. Figure 1 illustrates the process of exosome biogenesis [44, 45, 46].

The process of exosome formation begins with the inward budding of the plasma membrane, leading to the internalization of soluble extracellular molecules together with membrane‐associated proteins, which ultimately results in the formation of early sorting endosomes (ESEs). These ESEs serve as the first compartment of the endocytic pathway, providing the structural framework and molecular constituents for the later formation of exosomes [47]. For example, during erythrocyte maturation, reticulocytes endocytose gold‐labeled transferrin, directing it to early endosomes, which considered a hallmark of the initial phase of exosome biogenesis [45, 48].

As early endosomes undergo maturation, their limiting membranes undergo inward invagination, leading to the formation of MVBs [49]. Specific molecules and particles are sequestered and enclosed within intraluminal vesicles inside MVBs, a crucial step in defining the cargo and eventual function of exosomes [50]. Key factors that regulate this process include the endosomal sorting complex for transport, apoptosis‐associated proteins such as ALIX, and tetraspanins like CD81, CD9, and CD63 [44, 51].

After the formation of MVBs, they can either fusion with lysosomes for degradation or fuse with the plasma membrane, allowing the release of intraluminal vesicles into the extracellular space as exosomes. The process of exosome release is tightly regulated by small GTPases of the Rab family, such as Rab27a/b and Rab7, as well as the soluble N‐ethylmaleimide‐sensitive factor attachment protein receptor complex. For example, Rab27a/b promotes the fusion of MVBs with the plasma membrane by through interactions with the SNARE complex, thereby facilitating the release of exosomes into the extracellular environment [52, 53].

#### 2.1.2 The Composition of Exosomes

Exosomes are characterized by their complex composition and functional diversity, encapsulating bioactive molecules such as proteins, nucleic acids, metabolites, and lipids [54, 55]. Table 1 summarizes the various proteins involved in exosome biology and functions. These molecules are selectively incorporated into exosomes through precisely regulated molecular processes that are crucial for facilitating intercellular communication. The protein constituents of exosomes can be classified into two primary categories. The first group comprises proteins involved in exosome biogenesis, cargo sorting, membrane budding, and vesicle release. These include cytoskeletal components, endosomal sorting complex required for transport‐associated proteins (e.g., ALIX, TSG101), tetraspanins (e.g., CD9, CD63, CD81), and heat shock proteins (e.g., HSP70, HSP90). These proteins not only maintain the structural stability of exosomes but also regulate their formation and assist in determining their cellular destinations [56]. Additionally, Rab GTPases (e.g., Rab27a/b, Rab11) [57] and SNARE [52] proteins contribute to vesicle docking and fusion events, further influencing exosome secretion dynamics. These proteins are essential for the formation, secretion, structural stability, and precise targeting of exosomes. The second group consists of cell‐specific proteins inherited from the parental cells, reflecting the physiological or pathological state of their origin. These include immune‐related molecules such as MHC class I and II, costimulatory molecules (e.g., CD86), and cell‐ or tissue‐specific markers such as platelet‐derived factors (e.g., CD41a, von Willebrand factor), neuronal proteins (e.g., L1CAM), or tumor‐associated proteins (e.g., transforming growth factor‐β [TGF‐β], EGFRvIII). These proteins are functionally relevant in mediating immune responses, modulating the tumor microenvironment, and facilitating tissue‐specific targeting of exosomes [58].

**TABLE 1:** Proteins involved in exosome biology and their functions.

Category	Examples	Function	References
Tetraspanins	CD9, CD63, CD81, CD82, CD53	Play roles in vesicle formation, membrane organization, and cell communication	[59, 60]
ESCRT machinery/MVB biogenesis	ESCRT‐0, I, II, III; ALIX, VPS4, TSG‐101, Gag	The machinery helps sort cargo, form MVBs, release exosomes, and reshape membranes.	[61, 62]
Heat shock proteins	Hsp70, Hsp90, Hsc70, Hsp60, Hsp20, Hsp27	Involved in exosome release and signal transduction, stabilizing proteins during exosome biogenesis and secretion	[63]
Membrane transport and fusion	Rab GTPases, annexins, dynamin, syntaxin, AP‐1, Arp2/3, SNAP	Key for exosome formation, transport, and membrane fusion, especially Rab5 and Rab7 in sorting and release	[64]
Antigen presentation	MHC class I, MHC class II, CD86	Helps present antigens and activate T‐cells in immune responses	[65, 66]
Cytoskeletal proteins	Actin, vimentin, talin, ezrin, tubulin, cofilin, moesin	Maintains vesicle integrity and helps form and release exosomes	[54]
Adhesion	P‐selectin, CD146, CD166, ICAM‐1, ALCAM, MAC‐1, integrin α chain, integrins α4 β1, LFA‐3, CD53, CD326, CD11a, CD11b, CD11c, MFG‐E8/lactadherin	Helps exosomes attach to target cells for uptake and immune response	[67]
Glycoproteins	β‐Galactosidase, O‐linked glycans, N‐linked glycans	Located on exosome surfaces, aiding targeting and uptake by cells	[68]
Growth factors and cytokines	TNF‐α, TGF‐β, TRAIL	Involved in exosome signaling and immune response, affecting inflammation and tumors	[69]
Signal transduction	Erk2, Fyn, RhoA, catenin, syntenin, LCK	Key for exosome signaling, affecting cell functions and pathways	[70, 71]
Enzymes	Peroxiredoxin 1, fatty acid synthase, pyruvate kinase, ATP citrate lyase	Helps select exosome cargo and supports cell metabolism and growth	[72]
Other signaling receptors	FasL, TNF receptor, transferrin receptor	Present in exosomes, involved in immune and apoptotic signals	[73]
Antiapoptosis	ALIX, thioredoxine peroxidase	Helps prevent cell death and controls signals during exosome transport	[74, 75]
Lipid rafts	Flotillin‐1, cholesterol, LBPA, stomatin	Important for exosome membranes, helping formation, cargo sorting, and stability	[76]
Miscellaneous	Histone 1, 2, 3, clathrin, ferritin light chain 1 and 2, CD18, CD147, complement factor 3, CD55, CD59	Contains proteins for cargo selection, exosome transport, immune response, and cell signaling	[45]

Abbreviations: ALCAM, activated leukocyte cell adhesion molecule; ALIX, ALG‐2‐interacting protein X; AP‐1, adaptor protein complex 1; Arp2/3, actin‐related protein 2/3 complex; ATP, adenosine triphosphate; CD, cluster of differentiation; Erk2, extracellular signal‐regulated kinase 2; ESCRT, endosomal sorting complex required for transport; Ezrin, ezrin–radixin–moesin (ERM) family member; Gag, group‐specific antigen; Hsc, heat shock cognate protein; Hsp, heat shock protein; ICAM‐1, intercellular adhesion molecule 1; LBPA, lysobisphosphatidic acid; LCK, lymphocyte‐specific protein tyrosine kinase; LFA‐3, lymphocyte function‐associated antigen 3; MAC‐1, macrophage‐1 antigen (CD11b/CD18); MCH, major histocompatibility complex; RhoA, Ras homolog family member A; SNAP, soluble NSF attachment protein; TGF‐β, transforming growth factor beta; TNF‐α, tumor necrosis factor alpha; TSG‐101, tumor susceptibility gene 101; VPS4, vacuolar protein sorting‐associated protein 4.

In contrast, ectosomes (also known as microvesicles), which are shed directly from the plasma membrane, are typically enriched in plasma membrane‐associated proteins such as integrins and selectins and often display externalized phosphatidylserine (PS) [77]. However, due to their direct budding from the cell surface, ectosomes generally exhibit less selective molecular packaging compared with exosomes. This selective cargo loading process confers exosomes with superior molecular specificity and functional precision, making them particularly advantageous for intercellular communication and therapeutic delivery applications.

Exosomes also contain diverse nucleic acids, such as messenger RNA (mRNA), microRNA (miRNA), long noncoding RNA (lncRNA), and circular RNA (circRNA), all of which play essential roles in the regulation of intercellular gene expression. By facilitating exosome‐mediated transfer, these nucleic acids can profoundly influence gene expression patterns in recipient cells, regulating cellular processes at transcriptional, posttranscriptional, and translational levels. For example, miRNAs, which are key nucleic acid components within exosomes, regulate gene expression in recipient cells by targeting specific mRNAs and inhibiting their translation. Moreover, novel noncoding RNAs, including lncRNAs and circRNAs, also play crucial roles in exosome‐mediated intercellular communication [78].

Exosomes are similarly enriched with a range of metabolites and lipids, essential for energy transfer and intercellular signaling. Lipids, as integral structural components of the exosome membrane, not only confer the exosome's physical properties but also facilitate the fusion process with the membranes of recipient cells [79]. Additionally, metabolites contained within exosomes, such as amino acids, sugars, and nucleotides, act as key mediators of intercellular energy transfer and metabolic regulation. Via exosomal transfer, these metabolites can alter the metabolic state of recipient cells, thereby modulating their biological functions.

#### 2.1.3 The Biological Functions of Exosomes

Exosomes exhibit remarkable biological functions, particularly their ability to cross cellular membranes and regulate diverse physiological and pathological processes through mechanisms including endocytosis, receptor–ligand interactions, and membrane fusion [47]. Exosomes serve as pivotal mediators of intercellular communication by facilitating the transfer of bioactive molecules such as proteins, nucleic acids, and metabolites to recipient cells, subsequently regulating their physiological functions. A representative example within the nervous system involves neuron‐derived exosomes that transport specific miRNAs to astrocytes, inducing functional modifications in these glial cells and playing an essential role in the regulation of neural homeostasis [80]. This process encompasses not only the delivery of genetic material but also the regulation of cellular processes.

In the nervous system, exosomes are crucial for processes such as neuronal proliferation, differentiation, and maturation, and they also play a significant role in immune modulation and neuroprotection. For instance, exosomes originating from neural stem cells (NSC‐Exo) enhance neuronal differentiation and accelerate maturation through the delivery of miR‐9. Likewise, the miRNAs present in exosomes released by hypothalamic NSCs play a crucial role in influencing the aging process [81].

Furthermore, these exosomes transport neurotrophic factors, such as brain‐derived neurotrophic factor (BDNF) and nerve growth factor, which are vital for the survival and development of neurons [82, 83]. In the context of immune modulation, exosomes convey molecules such as immune‐related proteins and miRNAs that modulate the activity of immune cells. For example, exosomes derived from ReNcell VM cells exhibit strong anti‐inflammatory effects by suppressing the mitogen‐activated protein kinase signaling pathway in BV2 cells. Immune‐related proteins found in NSC‐derived exosomes, such as heme‐binding protein and netrin‐1, further bolster their roles in neuroregeneration and immune regulation. These actions highlight the critical involvement of exosomes in regulating inflammatory responses, autoimmune diseases, and tumor immunity [84].

Due to their critical involvement in mediating intercellular communication and regulating biological activities, exosomes present significant therapeutic potential for addressing various diseases. For instance, exosomes derived from pericytes offer promising therapeutic avenues for neurodegenerative disorders by delivering miRNAs and inflammatory mediators [85]. Similarly, exosomes from umbilical cord mesenchymal stem cells (MSCs) show strong potential for treating cardiovascular diseases, including myocardial fibrosis [86]. These findings underscore that targeted manipulation of exosome composition and function can facilitate effective therapeutic interventions across a wide range of diseases.

The variability in exosome composition and function is profoundly shaped by the physiological state of the donor cells and surrounding environmental factors. The “parental exosome signature,” which mirrors the characteristics of donor cells, and microenvironmental conditions such as cellular aging and oxygen levels during culture, significantly influence exosome properties [87]. Therefore, optimizing these conditions can improve the efficacy of exosome‐based therapeutic strategies.

**FIGURE 2:**
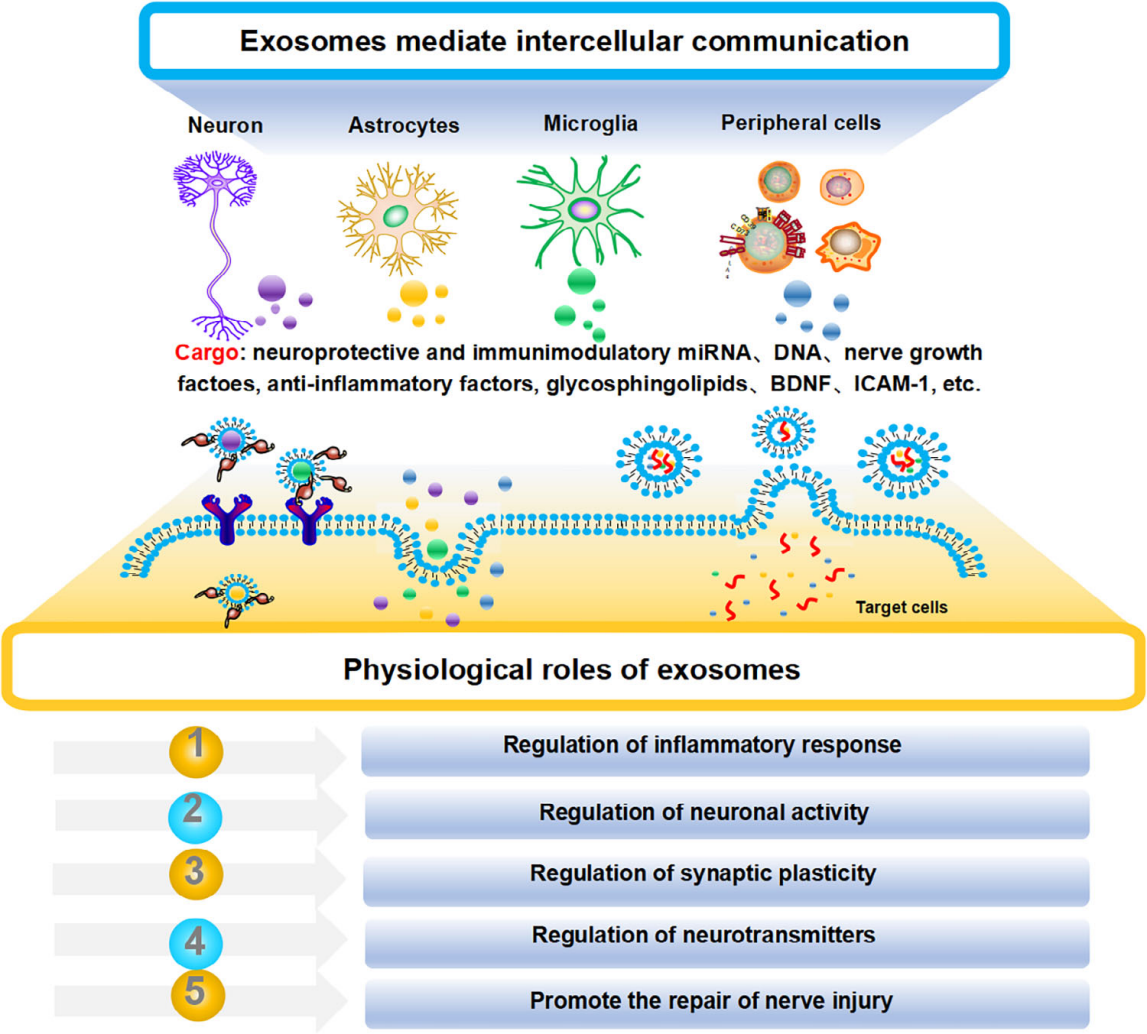
The biological roles and functions of exosomes. Exosomes encapsulate a wide array of bioactive molecules, particularly miRNAs, which play crucial roles in supporting neurodevelopment. These bioactive molecules interact with target cells, influencing signal transduction pathways and modulating gene expression. Through such mechanisms, exosomes help regulate inflammatory responses, reduce oxidative stress, modulate neuronal functions, and maintain neurotransmitter equilibrium. Additionally, they are essential in promoting neural repair following injury. Gaining deeper insights into the bioactive components of exosomes in the context of neurodevelopment will provide valuable understanding of the complex regulatory networks governing intercellular communication. miRNAs, microRNAs; ICAM‐1, Intercellular adhesion molecule 1; BDNF, brain‐derived neurotrophic factor.

In summary, exosomes possess distinct compositional and structural features that vary depending on their production conditions, which directly affect their biological functions, including targeting precision, biodistribution, biocompatibility, compartmentalization, permeability, and biodegradability, as illustrated in Figure 2 [17]. Exosome‐mediated intercellular communication facilitates the transfer of various biomolecules, exerting paracrine effects at both local and systemic levels [88, 89].

### 2.2 Exosome Isolation Techniques

**FIGURE 3:**
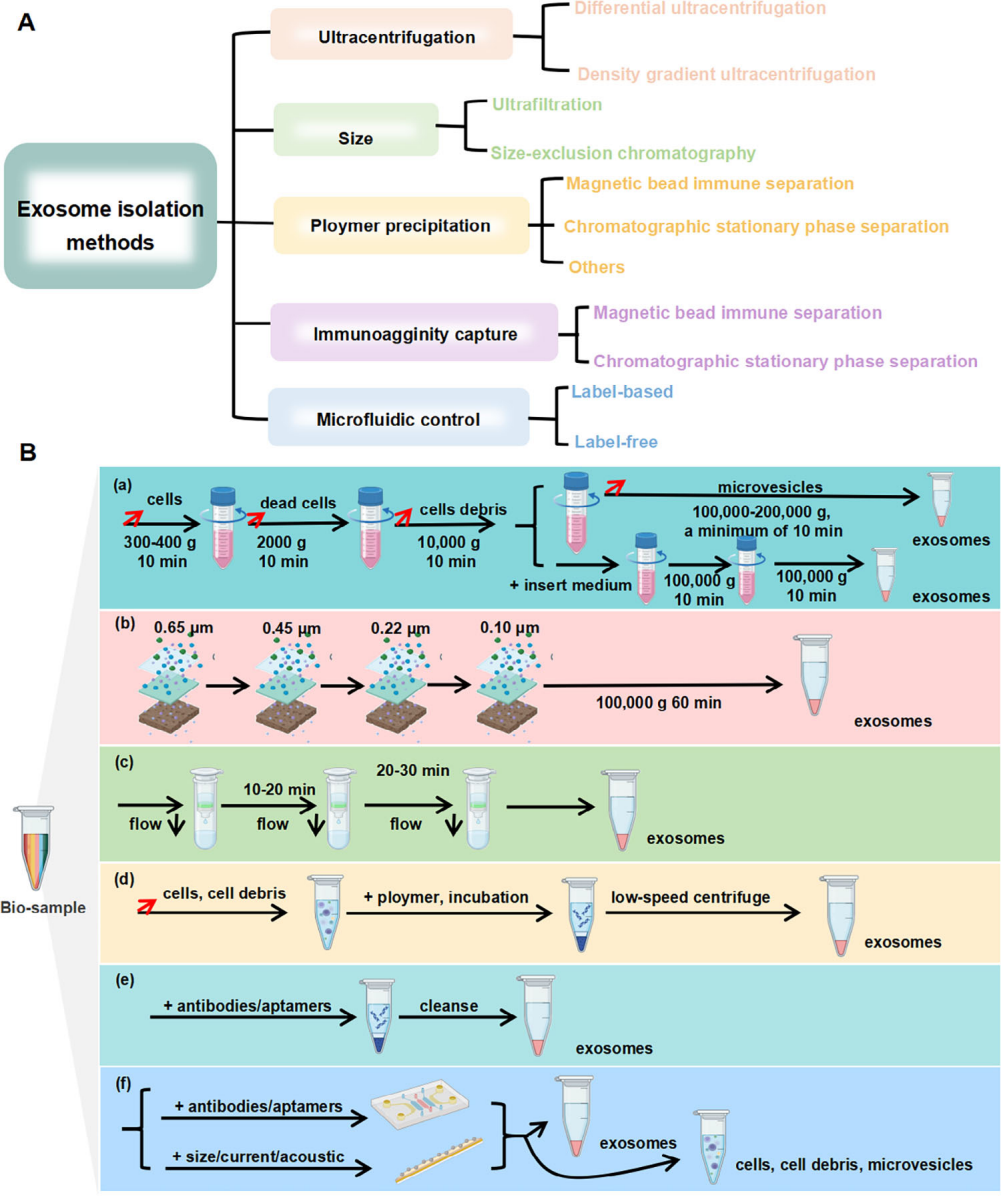
Classification of exosome isolation methods and schematic diagram of isolation principles. (A) Classification of exosome isolation methods. (B) Schematic diagram of isolation principles of exosome isolation methods.

The isolation and enrichment of exosomes are crucial for driving forward both foundational research and clinical innovations. Nonetheless, their nanoscale dimensions and low buoyant density present considerable obstacles to their effective separation and purification from complex biological matrices. Despite these obstacles, considerable advancements have been achieved by researchers through the application of various methodologies, including ultracentrifugation, ultrafiltration, size‐exclusion chromatography (SEC), polymer precipitation, affinity‐based capture, and microfluidic technologies (Figure 3A). Numerous laboratories have successfully implemented these techniques; however, the resulting exosome preparations can vary significantly in yield (i.e., the total amount of exosomes recovered), purity (i.e., the proportion of exosomes relative to coisolated contaminants such as proteins or other vesicles), and concentration (i.e., the number of proteins, nucleic acids, and other biomolecules per unit volume). This section offers a detailed analysis of exosome isolation methodologies grounded in distinct principles (Figure 3B), systematically comparing their respective strengths and limitations to guide researchers in selecting the optimal approach for their particular scientific aims. Effective isolation methods are fundamental to advancing the broad application of exosomes, especially in the context of therapies targeting CNS disorders that require traversal across the BBB (Table 2).

**TABLE 2:** Comparison of various exosome isolation techniques.

Isolation method	Principle	Advantages	Limitations	Applications	References
Differential ultracentrifugation	Stepwise centrifugal separation by size and density	Gold standard, widely used, high efficiency, minimal biochemical interference	High cost, time consuming, unsuitable for small samples, potential RNA/DNA degradation at high speeds	Large‐volume samples, studies requiring intact biomolecular components	[89, 90]
Density gradient ultracentrifugation	Uses density gradient media (e.g., sucrose, iodixanol) to separate exosomes based on density	High purity, good for precise density‐based separation	Complex, time consuming, low throughput, potential lipoprotein contamination	Exosome isolation from complex matrices (e.g., plasma, virus‐infected samples)	[91, 92]
Size‐based isolation (ultrafiltration, SEC)	Size‐based filtration or chromatography to isolate exosomes from other particles	Simple, reproducible, scalable, avoids chemical contamination	Membrane clogging, damage, contamination risk, reduced purity when used alone	Large sample volumes, combined with other techniques (e.g., SEC + ultracentrifugation) to enhance exosome purity	[93, 94, 95]
Polymer precipitation	Uses polymers (e.g., PEG) for exosome precipitation via hydrophilic/hydrophobic interactions	Simple, rapid, cost‐effective, preserves exosome integrity	Lower purity, unsuitable for high‐purity isolation, risk of coprecipitation with contaminants	Large‐scale isolation, enrichment from biofluids, cost‐effective for clinical and industrial applications	[96, 97, 98, 99, 100]
Immunoaffinity capture	Targets surface proteins (e.g., CD9, CD63, CD81) using antibodies or aptamers for selective exosome capture	High specificity, recovery, and bioactivity retention	High cost, complex protocols, contamination risk, limited throughput	Clinical diagnostics, personalized medicine, cancer research, requiring precise exosome separation	[101, 102, 103, 104, 105]
Microfluidics‐based isolation	Separation based on physical properties (size, dielectric, surface markers) in microfluidic devices	High efficiency, automation, integration with downstream analysis, low sample volume, label‐free options	High setup cost, complex operation, developmental for large‐scale applications	Point‐of‐care diagnostics, small sample volumes, personalized medicine, real‐time monitoring	[106, 107, 108, 109]

Abbreviations: CD, cluster of differentiation; SEC, size‐exclusion chromatography.

#### 2.2.1 Exosome Isolation Based on Ultracentrifugation

Ultracentrifugation is a well‐established and extensively used technique for isolating exosomes, leveraging high centrifugal forces to concentrate and sediment them at the base of the centrifuge tube [110, 111, 112]. This methodology primarily consists of two distinct approaches: differential ultracentrifugation and density gradient ultracentrifugation (Figure 1B‐a). Differential ultracentrifugation is often viewed as the “gold standard” for exosome isolation because of its high efficiency and wide applicability [113, 114]. By progressively increasing centrifugal forces to achieve efficient separation, this method takes advantage of the size and density differences among cellular components, such as cells, cell debris, apoptotic bodies, and exosomes [115, 116]. The process begins with a low‐speed centrifugation step (approximately 300–400 g) to remove the bulk of cells and debris, followed by an intermediate‐speed centrifugation (approximately 10,000×*g*) to further purify the sample by clearing residual contaminants [117]. Ultimately, high centrifugal forces (100,000–200,000×*g*) are employed to pellet the exosomes [118]. This method minimally disrupts the biochemical integrity of exosomes and is particularly suited for processing large‐volume samples. However, several limitations persist, including its inefficiency for small sample volumes, the high cost of equipment, labor‐intensive protocols, and the possibility of degradation of the integrity of exosomal contents, such as RNA and DNA, during high‐speed centrifugation.

Density gradient ultracentrifugation represents a more advanced version of differential ultracentrifugation, involving the use of an inert medium, such as sucrose or iodixanol, to generate a defined density gradient [119, 120]. This setup facilitates the migration to their optimal buoyant density zone during the centrifugation process. This technique is particularly effective for density‐dependent separation, with exosomes typically concentrating within the 1.13–1.19 g/mL range. For exosome isolation from serum‐free media of human MSCs, Gupta et al. [121] made a comparison between traditional differential ultracentrifugation and a one‐step sucrose cushion technique. The protocol began with low‐speed centrifugation (300×*g* for 10 min) to remove cellular debris, followed by medium‐speed centrifugation (10,000×*g* for 30 min) to eliminate microvesicles, and concluded with ultracentrifugation using a sucrose cushion. This approach significantly enhanced exosome recovery, as verified by nanoparticle tracking analysis (NTA) and transmission electron microscopy (TEM) [122, 123].

Raj et al. [93] investigated exosome isolation from urine by integrating multistep differential ultracentrifugation with a two‐layer sucrose/D_2_O cushion, effectively removing denser vesicular contaminants and thereby enhancing exosome purity. Iodixanol‐based density gradient centrifugation demonstrated several advantages over sucrose density gradients, such as reduced viscosity, metabolic inertness, low cytotoxicity, and superior preservation of cellular integrity and functionality. Li et al. [94] employed an iodixanol‐buffered density gradient platform with a 60% iodixanol cushion, significantly improving exosome recovery, preserving their structural integrity and biological activity, and efficiently eliminating protein contaminants and nonexosomal nanoparticles. The high biochemical inertness and compatibility of iodixanol render it an optimal choice for biochemical and physiological investigations. Research by Konadu et al. [95] further highlighted the utility of iodixanol density gradient ultracentrifugation for isolating complex biological samples, including particles derived from the plasma of HIV‐1‐infected patients. This technique successfully discriminated exosomes from viral particles on the basis of density. Although density gradient ultracentrifugation significantly enhances exosome purity, it still presents several challenges, such as operational complexity, high technical demands, low throughput, lengthy processing times, and the inability to completely eliminate lipoproteins and lipid contaminants, necessitating further optimization for practical applications [96].

#### 2.2.2 Size‐Based Isolation Methods

Size‐based isolation methods primarily include ultrafiltration (Figure 1B‐b) and SEC (Figure 1B‐c), offering distinct yet complementary approaches for exosome separation. Ultrafiltration utilizes membrane filters with specific molecular weight cutoffs. These include sequential filtration, serial filtration, centrifugal ultrafiltration, and tangential flow filtration, to achieve size‐dependent exosome isolation [97]. This technique can be performed at ambient temperatures while minimizing the risk of chemical contamination; however, it remains susceptible to challenges such as membrane fouling and filter degradation.

In contrast, SEC facilitates exosome separation and purification by capitalizing on size disparities between exosomes and the porous gel matrix. This approach is straightforward, highly reproducible, and particularly well suited for handling large sample volumes [118]. A significant body of research has shown that integrating SEC with other methods, such as ultracentrifugation, markedly improves the efficiency, yield, and purity of exosome isolation. For example, Koh et al. [124] developed an approach combining plasma pretreatment, ultracentrifugation, and SEC, yielding highly reproducible and efficient exosome isolation from large sample volumes. Similarly, Yang et al. [98] demonstrated that custom SEC columns provided superior purity and yield, further validating the overall efficacy of this technique when compared with ultracentrifugation and commercial kits.

Nonetheless, SEC alone has inherent limitations, often requiring integration with other techniques, such as ultracentrifugation, to achieve optimal separation outcomes. In general, combined methodologies are more effective in enhancing the quality and purity of exosomes isolated from serum. Despite these advancements, challenges such as insufficient purity and residual contaminants may still arise [118]. In conclusion, size‐based isolation techniques offer robust strategies for exosome separation and purification, though additional refinement is necessary to further improve overall efficiency.

#### 2.2.3 Polymer Precipitation‐Based Isolation Techniques

Polymer precipitation has been widely utilized for over half a century as a robust method for the enrichment and purification of viruses and related nanoparticles, including exosomes (Figure 1B‐d). This technique exploits hydrophilic and hydrophobic interactions between polymers and target particles in the sample, creating a microenvironment conducive to particle precipitation. PEG, a commonly used polymer, has proven highly effective in boosting both exosome concentration and recovery [99, 125]. Ludwig et al. [126] optimized the PEG precipitation method by introducing additional washing and ultracentrifugation steps, which significantly enhanced exosome purity.

In addition, thermo‐responsive technologies have been developed, including poly (N‐isopropylacrylamide‐co‐N‐acryloyloxysuccinimide) copolymers, which facilitate controllable exosome aggregation and release through temperature‐induced phase transitions. This innovation highlights the promise of stimulus‐responsive separation systems [101]. In food science and biomedicine, this method has been effectively employed to extract and purify exosomes from bovine milk, facilitating progress in dairy cow health management and DDSs [102].

Polymer precipitation‐based exosome isolation techniques offer multiple advantages, including simplicity, efficiency, rapid processing, minimal equipment needs, and the preservation of exosome integrity. These attributes render this method particularly promising for both clinical research and commercial applications. Compared with traditional centrifugation methods, polymer precipitation not only reduces time and enhances reproducibility but is also valued for its ease of use and its ability to yield high‐purity vesicles containing small RNAs. Although this technique may not always produce completely pure exosomes, its capacity for rapid enrichment and concentration from large‐volume samples substantially reduces costs, making it well suited to both research and industrial applications [103].

#### 2.2.4 Immunoaffinity Capture‐Based Isolation Techniques

Exosome membranes are particularly enriched with tetraspanins, including CD9, CD63, and CD81, rendering them ideal targets for accurate exosome isolation. By leveraging these membrane proteins, immunoaffinity‐based isolation techniques facilitate precise capture and enrichment of exosomes through antibody‐ or aptamer‐mediated immune interactions (Figure 1B‐e). These techniques primarily include magnetic bead‐based immunoisolation, solid‐phase chromatography‐based isolation, and various innovative immunoaffinity methodologies [127].

Magnetic bead‐based immunoisolation utilizes functionalized magnetic particles, including materials like iron, nickel, neodymium, or magnetite, in combination with targeted antibodies. This method utilizes magnetic forces to efficiently isolate exosomes from complex biological matrices, which is able to effectively mitigate matrix interference, thereby enhancing pre‐enrichment efficiency and detection sensitivity for exosomes [104]. For example, innovative strategies such as anion exchange magnetic beads and the Strep‐tag II‐based immunomagnetic isolation system have exhibited high recovery rates, increased purity, and the preservation of significant biological activity [128]. Furthermore, advancements such as photoactivated elution technology and biomimetic hedgehog‐structured magnetic beads have significantly improved isolation efficiency and biocompatibility [129].

Chromatography‐based solid‐phase isolation techniques, inspired by the principles of high‐performance liquid chromatography (LC), employ mechanisms such as hydrophobic interaction chromatography to efficiently isolate exosomes on solid phases, including polyester capillary‐channel polymer fibers. This approach not only enhances throughput and purity but also substantially decreases processing time and costs, and allows for excellent reusability across multiple isolation cycles [106]. The use of capillary‐channel polymer fiber micropipette tips has facilitated rapid and cost‐effective isolation from clinically relevant samples.

In addition to these conventional methods, Lee et al. [107] have developed a cost‐effective and user‐friendly point‐of‐care platform—paper‐based enzyme‐linked immunosorbent assay (ELISA). This platform employs streptavidin‐agarose bead immobilization technology to enable targeted detection of EVs and exosomes. This innovative methodology is particularly suited for resource‐limited regions or countries, demonstrating significant potential for widespread application. Conversely, Barati et al. [108] developed an innovative coaxial nanofiber structure, designed to enhance the efficient isolation of exosomes from bodily fluids. This structure consists of a polycaprolactone polymer core surrounded by an ultra‐thin (sub‐10 nm) gelatin shell, which is temperature‐sensitive, enabling effective exosome release at near‐physiological temperatures (37°C), thereby minimizing contamination during isolation and preserving exosome integrity.

This design presents significant potential for the efficient and specific capture of exosomes from complex biological fluids. These platforms improve exosome isolation through immobilization techniques and micro–nano structural designs, delivering cost‐effective and user‐friendly exosome separation, particularly appropriate for resource‐limited environments. The temperature‐sensitive release mechanism of coaxial nanofiber structures, combined with their high surface area, further enhances exosome capture efficiency and purity. Although this technique provides high specificity for exosome isolation, it is generally more expensive and time consuming than other methods. Moreover, the limited availability of high‐quality antibodies can further restrict its broader application [130, 131]. The process typically involves immobilizing antibodies that target exosome‐specific markers onto a solid‐phase substrate. The exosome‐containing extracellular fluid is then incubated with this substrate, allowing exosomes to bind through specific antigen–antibody interactions [17, 132].

#### 2.2.5 Microfluidics‐Based Isolation Techniques

Microfluidic technology has demonstrated significant potential and advantages in the field of exosome isolation, primarily through both affinity‐based (labeled) and label‐free methodologies (Figure 1B‐f) [133, 134, 135, 136]. In labeled isolation techniques, antibodies or magnetic beads are utilized to target specific biomarkers on exosomes (e.g., CD9, EpCAM, CD63) through immunocapture mechanisms, thereby facilitating the efficient isolation of exosomes from complex biological samples. For instance, the novel herringbone‐groove microfluidic device developed by Hisey et al. [137] and the immunomagnetic bead technology introduced by Tayebi et al. [138] have yielded high‐purity and high‐recovery separation outcomes. Integrated platforms, including the EXID system and ExoSD chip, have further optimized isolation efficiency and detection precision, thereby offering robust support for personalized medicine and cancer diagnostics. Nevertheless, despite improved separation performance, labeled methods continue to face challenges such as elevated costs, operational complexity, and the potential for sample contamination.

Conversely, label‐free isolation techniques leverage the physical properties of exosomes (e.g., size and dielectric characteristics), offering advantages such as reduced costs, simplicity, and rapid processing times. Examples of these techniques include ultrafiltration technology developed by Chen et al. [139], microfluidic devices designed by Yang et al. [140], and the dual tangential flow filtration system introduced by Hua et al. [141], all of which effectively isolate exosomes by modulating membrane pore sizes or applying physical force fields. Notably, the angiotensin‐converting enzyme microarray chip and dielectrophoresis techniques have facilitated rapid exosome isolation and biomarker analysis from small sample volumes. Emerging technologies, such as acoustofluidic platforms that integrate acoustic and microfluidic principles, enable label‐free, contactless isolation of exosomes from whole blood, demonstrating significant potential for widespread application.

Overall, microfluidic technology has not only enhanced the efficiency and purity of exosome isolation but has also facilitated the integration and automation of the isolation process. To further advance this field, it is imperative to optimize isolation strategies, reduce costs, increase processing throughput, and minimize sample contamination. Microfluidic platforms are expected to take on a more prominent role in exosome research, disease diagnostics, and drug development as technology continues to evolve [142].

### 2.3 Characterization of Exosomes

The characterization of exosomes is a crucial aspect in elucidating their biological properties, functions, and prospective applications in drug delivery and clinical therapeutics. The absence of standardized identification protocols further limits reproducibility and hampers cross‐study comparisons. To address these issues, the MISEV‐2023 guidelines recommend an integrative approach using complementary quantitative techniques [143]. The application of a diverse array of techniques facilitates a thorough description of the physical properties of exosomes, encompassing size, concentration, and morphology, and enables a detailed analysis of their molecular composition and functional characteristics. This multifaceted methodology provides a solid foundation for the implementation of exosomes in various medical applications [144].

#### 2.3.1 Single EVs Analysis

TEM and scanning electron microscopy offer high‐resolution images of exosomal morphology owing to their exceptional magnification and resolution; however, the sample preparation process may impact the resultant images. Cryo‐electron microscopy, which utilizes rapid freezing in liquid nitrogen, significantly enhances imaging accuracy by preserving the native architecture of exosomes. Additionally, atomic force microscopy, which performs nanoscale scanning of exosomal surfaces, provides noninvasive, high‐resolution imaging of surface structures and mechanical properties, thus making it an invaluable tool in the characterization of exosomes [145, 146]. Nevertheless, characterization of exosomes based solely on morphological parameters remains insufficient, necessitating a more comprehensive analysis incorporating additional parameters.

Currently, several methods can measure vesicle concentration, each with pros and cons [147, 148]. NTA tracks vesicle movement to estimate concentration and size, detecting small differences. However, it may overestimate concentration in the presence of protein aggregates or large particles and needs careful calibration [148]. Resistive pulse sensing (RPS) measures size and concentration by detecting changes in electrical current as vesicles pass through a nanopore. Although RPS is sensitive and accurate, it requires complex sample prep, equipment, and can be difficult to analyze [149].

Characterizing the physical properties of exosomes typically involves optical techniques, including NTA, RPS, flow cytometry, dynamic light scattering (DLS) and multiangle light scattering, to assess size distribution [150, 151]. DLS detects light scattering signals from particles, demonstrating efficacy in analyzing sizes ranging from 1 nm to 6 µm; however, its performance may be diminished in complex suspensions. Furthermore, although DLS can simultaneously assess the size distribution of all particles within a sample, it has inherent limitations in accurately quantifying particle concentration [152]. Conversely, NTA tracks the Brownian motion of individual nanoparticles using microscopy, thereby enabling the precise determination of exosome size, distribution, and concentration, particularly within the range of 10 nm to 2 µm. The fluorescence detection capabilities of NTA further augment its accuracy and versatility when analyzing complex samples.

Flow cytometry provides sensitive, multiparametric, and quantitative analysis of exosomes but has limitations, including size detection thresholds, standardization challenges, background noise, and complex sample preparation. It operates in two modes: bead‐based and single vesicle. Bead‐based flow cytometry captures exosomes on fluorescent beads coated with specific antibodies, complicating sample preparation [153]. In contrast, single vesicle flow cytometry analyzes individual exosomes directly without beads, using specialized instruments to accurately measure size and concentration [154].

#### 2.3.2 Characterization of EVs Content and Cargo

The analysis of the biochemical composition of exosomes is facilitated by the integration of proteomics and mass spectrometry (MS), enabling the identification and quantification of specific exosomal proteins and providing insight into their roles in intercellular communication [143]. Techniques such as western blotting [155], flow cytometry [156], and ELISA are essential for the detection of exosomal molecular markers. Notably, flow cytometry has enhanced the direct analysis of small exosomes with the advantage of bead‐based techniques, thereby enabling precise identification of their cellular origins and functions. Moreover, ELISA has exhibited considerable potential for the specific analysis of cancer‐associated exosomes. High‐throughput sequencing [157] focuses on the RNA content of exosomes, shedding light on their unique roles in the transfer of genetic information. Lipidomics, utilizing LC–MS [158], has yielded a comprehensive characterization of the lipid composition of exosomes, thereby enhancing our understanding of their roles in cellular signaling and energy regulation.

Functional analyses of exosomes are typically performed through a variety of bioactivity assays designed to assess their effects on recipient cells, including the modulation of cell proliferation, migration, and differentiation. Fluorescently labeled exosomes, employed in cellular uptake and tracking experiments, allow researchers to monitor interactions between exosomes and target cells, as well as their intracellular trafficking dynamics. These investigations offer critical insights into the biological functions of exosomes and highlight their potential applications in drug delivery and therapeutic interventions. Furthermore, these studies contribute to the optimization of DDSs, enhancing therapeutic efficacy and minimizing associated side effects.

### 2.4 Exosomes as Promising Brain Drug Carriers

**FIGURE 4:**
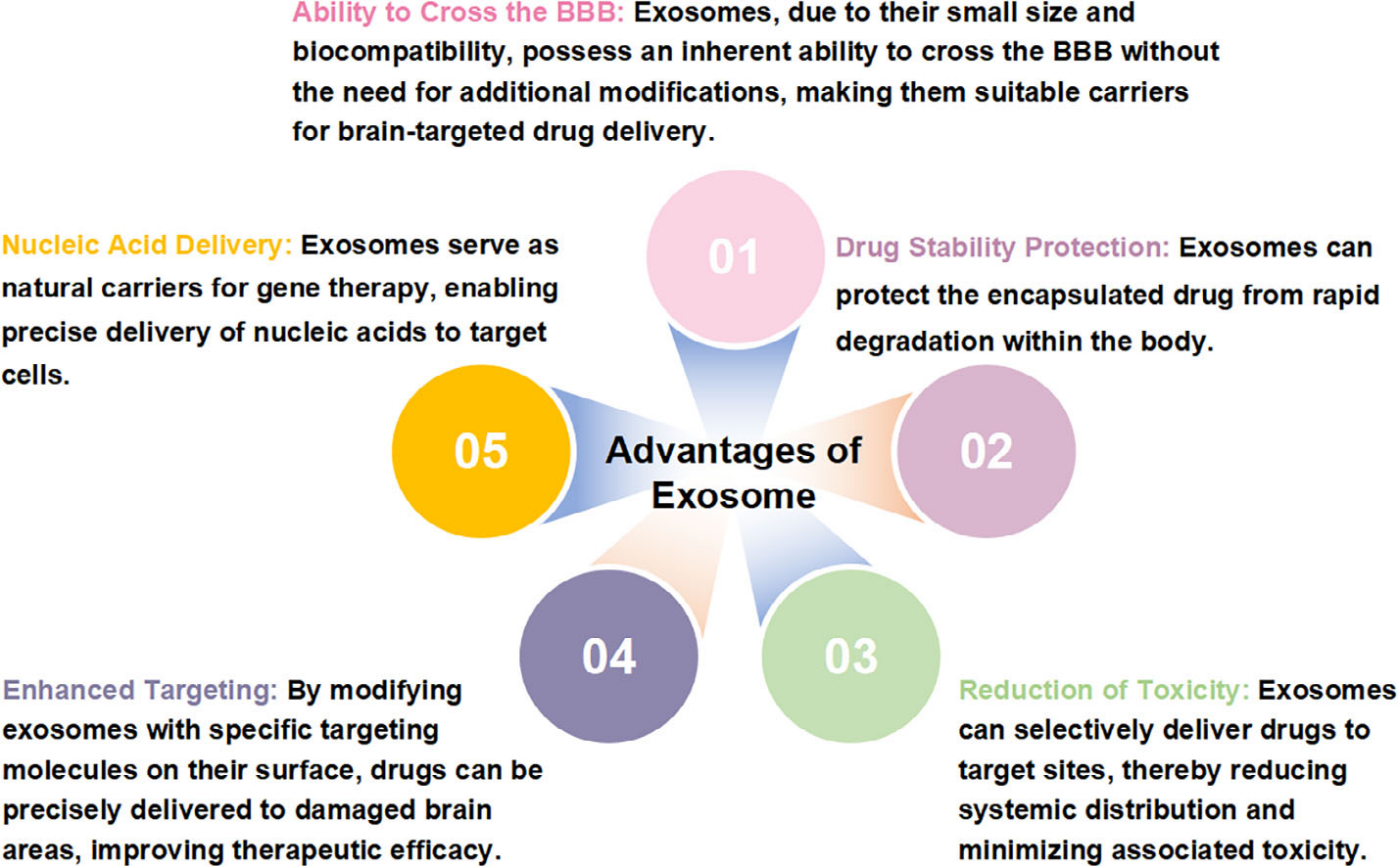
Advantages of exosomes. Exosomes, as naturally occurring carriers for therapeutic delivery, offer numerous advantages over synthetic delivery methods.

In recent years, researchers have made significant strides in the design and development of nanocarrier systems, optimized for efficient traversal of the BBB with high efficiency and deliver therapeutic agents with precision to targeted brain regions. Notwithstanding these innovations, synthetic nanoparticles laden with therapeutic agents still face three significant challenges: the risk of potential toxicity, rapid clearance by the MPS during circulation, and limited tissue‐selective distribution [159]. Within this paradigm, exosomes, which are nanoscale EVs actively secreted by multiple cell types, demonstrate dual clinical significance as both reliable diagnostic biomarkers for NDs and potential therapeutic vehicles for advanced DDSs. Researchers have actively investigated cellular engineering techniques to modify the surfaces of exosomes or augment their nucleic acid cargo, thereby enhancing their functionality in the realm of nanobiotechnology, particularly in facilitating the crossing of the BBB as a targeted drug delivery platform [160]. As illustrated in Figure 4, exosomes demonstrate significant advantages in circumventing the BBB, owing to their small size and inherent biological properties. Notably, even in the absence of surface modification, exosomes possess an intrinsic capability to traverse biological barriers, including the BBB. The subsequent sections will explore the numerous advantages of utilizing exosomes for therapeutic drug delivery applications.

#### 2.4.1 Improved Targeting

The intricate challenges posed by brain disorders emphasize the urgent need for therapeutic systems capable of precise targeting and efficient delivery strategies. In this context, exosomes, as naturally occurring carriers, have attracted significant attention due to their intrinsic therapeutic potential and remarkable targeting capabilities. For instance, while silybin (Slb) has shown promise in alleviating symptoms of AD, their capacity for precise brain targeting remains limited. Encapsulating Slb within macrophage‐derived exosomes (Exo‐Slb) markedly enhanced its binding affinity to Aβ monomers, reduced Aβ aggregation, and modulated astrocyte activation, thus improving cognitive function in AD mouse models [161]. Compared with synthetic carriers, such as PEGylated liposomes, which require extensive surface modification to achieve similar targeting, exosomes demonstrate natural receptor–ligand interactions that simplify delivery design [162].

Furthermore, although enkephalin has shown potential in enhancing neuronal survival, its clinical use is hindered by the restrictive nature of the BBB. Prior research has successfully overcome this challenge by encapsulating enkephalin in exosomes overexpressing transferrin receptors (TFRs), forming a “tarexo‐enkephalin” complex that efficiently crosses the BBB [163]. Although transferrin‐modified liposomes have been investigated to enhance BBB penetration, their uptake mechanisms are often less efficient and more prone to endosomal trapping compared with exosome‐mediated transport, which leverages endogenous transcytosis pathways [164]. In AD therapy, although corynoxine‐B is a biologically active compound, its absorption is significantly hindered by the BBB. Utilizing hippocampal neuron‐derived exosomes overexpressing Fe65 (Fe65‐EXO‐Cory‐B) facilitated the induction of autophagy in amyloid precursor protein‐expressing neurons, thereby promoting autophagy‐mediated therapeutic interventions in AD‐related neuronal cells [165]. In treating ischemic cerebrovascular conditions, exosomes engineered with the integrin αvβ3‐affinitive peptide demonstrated significantly improved targeting to ischemic lesion sites. When loaded with curcumin, these exosomes effectively suppressed inflammatory responses and apoptosis [166]. Although arginine–glycine–aspartic acid (RGD)‐functionalized polymeric micelles have shown similar targeting potential, exosomes possess endogenous surface integrins and tetraspanins that synergistically enhance targeting precision [167]. In addition, a groundbreaking study introduced a recombinant fusion protein composed of the RGD‐4C peptide and the PS‐binding domain of lactadherin (C1C2). This fusion protein demonstrated enhanced exosome targeting efficiency and led to a marked reduction in inflammation poststroke. Finally, magnetic nanovesicles, integrating iron oxide nanoparticles with MSC‐derived therapeutic growth factors, enabled precise ischemic injury targeting via magnetic guidance, resulting in enhanced anti‐inflammatory, angiogenic, and antiapoptotic effects, thereby significantly improving therapeutic outcomes [168]. While magnetic liposomes or polymeric carriers offer magnetic responsiveness, they lack the biological cargo complexity of MSC‐exosomes, which include growth factors, miRNAs, and cytokines with intrinsic therapeutic effects [38].

#### 2.4.2 Reducing Toxicity

Exosome‐based DDSs demonstrate exceptional capabilities to selectively target specific sites, thereby significantly minimizing the toxicity associated with freely circulating pharmaceuticals. A fundamental pathological characteristic of AD is mitochondrial dysfunction; however, existing mitophagy inducers that specifically target mitochondria are constrained by their inherent toxicity and suboptimal brain accumulation. A research team developed an innovative exosome‐based therapeutic system utilizing nanoscale EVs derived from MSCs (MSC‐EVs–SHP2), which were engineered to display elevated levels of the tyrosine phosphatase SHP2 on their surfaces. In a murine model of AD, MSC‐EVs–SHP2 demonstrated improved ability to penetrate the BBB, enabling the efficient delivery of SHP2 to the brain. This approach significantly promoted mitophagy in neuronal cells, reduced apoptosis induced by mitochondrial dysfunction, and inhibited the activation of the NLR family pyrin domain‐containing 3 (NLRP3) inflammasome, ultimately mitigating synaptic degeneration and cognitive impairment [169]. Furthermore, several studies have utilized the inherent brain‐targeting properties of blood‐derived exosomes, using a saturated solution incubation technique to effectively load dopamine into these vesicles. This innovative approach enhanced the distribution of dopamine in the brain, leading to better therapeutic effects in PD and notably decreasing the systemic toxicity typically linked to dopamine administration [170].

#### 2.4.3 Exosomes are Able to Enhance Drug Stability

Research conducted in both in vitro and in vivo settings have conclusively demonstrated that exosomes can effectively delay drug degradation. Furthermore, they preserve the stability of encapsulated therapeutics. Antisense oligonucleotides have shown considerable promise in attenuating α‐synuclein (α‐syn) expression, a critical target in the treatment of PD. However, antisense oligonucleotides face significant challenges, including limited membrane permeability and rapid degradation by proteolytic enzymes. The exosome‐mediated ASO4 delivery system (exo‐ASO4) facilitated efficient cellular uptake with minimal associated toxicity, resulting in a substantial reduction in α‐syn expression and aggregation, thereby improving dopaminergic neuron degeneration and enhancing motor function [171]. Additionally, the potential of antisense oligonucleotides is being explored as a novel therapeutic strategy for HD, which is recognized as an autosomal dominant neurodegenerative disorder. By employing an exosome‐based delivery system in conjunction with the self‐assembly of artificial genetic circuits, siRNA targeting mutant huntingtin (mHTT) was effectively delivered to the cortex and striatum, resulting in successful silencing of mHTT [172]. In another study, curcumin was encapsulated within exosomes that preserved specific functional traits from their original cells, such as the transport of lymphocyte function‐associated antigen 1 (LFA‐1) and intercellular adhesion molecule 1 (ICAM‐1). These exosomes effectively blocked Tau protein phosphorylation through the AKT/GSK‐3β signaling pathway, preventing neuronal death in both in vitro and in vivo models, which ultimately contributed to alleviating AD‐related symptoms [173].

In addition to enhancing drug stability, exosomes facilitate controlled release mechanisms. For example, exosomes loaded with curcumin and sourced from human endometrial stem cells demonstrated improved stability and prolonged release characteristics [174]. Exosomes isolated via ultrasound (termed Exo‐catalase) exhibited a high encapsulation efficiency and maintained the catalytic function of catalase. This method enabled a prolonged and controlled release, with catalase release rates remaining under 40% over a period of 24 h [175, 176]. Tom40, a pivotal mitochondrial membrane protein, is essential for enhancing mitochondrial function and safeguarding neurons against oxidative stress. In patients afflicted with NDs, Tom40 expression is markedly diminished. Nevertheless, delivering proteins into cells, especially across the BBB, poses a significant challenge. To address the challenge of delivering Tom40 across the BBB, researchers used the XPack lentivector system to engineer HEK293 cells that express Tom40 fused with a targeting peptide, facilitating its efficient encapsulation into exosomes. These exosomes were isolated using PEG–NaCl precipitation and confirmed to contain Tom40 by Western blot. Although the study was primarily in vitro, it leveraged the known ability of exosomes to cross the BBB via mechanisms such as receptor‐mediated and adsorptive‐mediated transcytosis (AMT). Their nanoscale size, lipid bilayer, and low immunogenicity further enhance brain‐targeting efficiency. The exosome‐mediated delivery of Tom40 promoted mitochondrial localization and protected neurons from oxidative stress, highlighting its therapeutic potential for NDs. The exosome‐mediated delivery of Tom40 has demonstrated protective effects against hydrogen peroxide‐induced oxidative stress in cells, underscoring its potential as a therapeutic approach for both AD and PD [177].

#### 2.4.4 Nucleic Acid Delivery

The efficient distribution of recombinant DNA to target cells continues to pose a substantial challenge within the realm of gene therapy. Recently, exosomes have emerged as natural carriers endowed with the capacity to precisely deliver therapeutic agents and biological materials to target cells [178]. Preliminary studies showed that after HEK‐293T cells and bone marrow MSCs were transfected to express miR‐29a and miR‐29b, the resulting mature miRNAs were encapsulated within exosomes generated by these cells. Subsequent research explored the potential of these modified exosomes in alleviating cognitive dysfunction in rat models of AD, characterized by impairments in spatial learning and memory. As a result, the administration of miR‐29‐containing exosomes into the rats effectively reversed these cognitive deficits [179].

In a related investigation, researchers assessed the exogenous loading of miR‐494 onto exosomes through reagent transfection. This loading was demonstrated to significantly reduce inflammation and neuronal apoptosis in affected regions. It also concurrently upregulated anti‐inflammatory factors, thereby exerting notable neuroprotective effects. Furthermore, exosomes loaded with miR‐494 facilitated behavioral recovery and enhanced axonal regeneration in the mice with spinal cord injury [179].

### 2.5 The Construction of Engineered Exosomes

#### 2.5.1 Exosome Drug Loading

Exosomes, functioning as DDSs, employ two principal strategies for drug incorporation: presecretion and postsecretion (Table 3).

**TABLE 3:** Different exosome drug loading methods and characteristics.

Loading mechanism	Loading method	Types of drugs	Advantages	Disadvantages	References
Presecretory loading	Incubation (CRISPR/Cas9)	Small molecule drugs; Nanomaterials	Simple; Little damage to exosomes; Wide applicability	Low loading efficiency; Cytotoxicity	[180, 181, 182, 183]
	Transfection	Proteins or peptides; Nucleic acids	Overexpression of desired; Molecules; Stability	Low loading efficiency; Cytotoxicity	[184, 185]
Postsecretory loading	Physical methods				
	Electroporation	Small molecule drugs; proteins or peptides; Nanomaterials; Nucleic acids	High loading efficiency	Exosome membrane damage; Exosome aggregation risk	[186, 187]
	Sonication	Small molecule drugs; Proteins or peptides; Nanomaterials	High loading efficiency	Exosome membrane damage; Exosome aggregation risk	[188]
	Freeze–thaw cycles	Proteins or peptides	Simple	Low loading efficiency; Exosome aggregation risk; Inactivation of proteins	[189]
	Extrusion	Small molecule drugs; Proteins or peptides	High loading efficiency	Exosome membrane damage; Exosome surface remodeling	[189]
	Chemical methods				
	Saponin‐assisted permeation	Nucleic acids	High encapsulation efficiency; Direct protein Incorporation	Hemolytic effect on blood cells	[190]
	Transfection	Nucleic acids	Overexpression of target molecules; Stability	Low loading efficiency	[191]
	Biological methods				
	Incubation	Small molecule drugs; Proteins or peptides; Nanomaterials; Nucleic acids	Simple; Inexpensive; Exosome integrity preservation	Contamination risk Stress on cells Low loading efficiency	[192, 193]
	Viral transduction	Nucleic acids	Preserves function of exosomes High encapsulation efficiency	Tedious; Laborious and safety risks	[194]

The presecretion strategy involves the initial introduction of drugs into donor cells, followed by their encapsulation during exosome biogenesis, primarily facilitated through techniques such as transfection and incubation. The CRISPR/Cas9 gene‐editing system represents a major advancement in cancer therapy due to its high specificity and therapeutic potential [180, 181]. However, its clinical application remains limited, primarily because of challenges such as immunogenicity. To overcome these barriers, engineered exosomes have been explored as effective delivery vehicles. When loaded with CRISPR/Cas9 components, exosomes can fuse with cancer cell membranes, enabling precise and noninvasive gene editing.

In contrast, the postsecretion strategy entails the direct introduction of drugs into preformed exosomes. Due to their unique structural characteristics, exosomes can encapsulate hydrophilic drugs within their aqueous core, whereas hydrophobic molecules are incorporated into their lipid bilayer. This method not only improves the in vivo stability of drugs but also optimizes their biodistribution. Nevertheless, considerable challenges persist in refining exosomes into highly efficient drug delivery vehicles. Drug loading techniques are commonly categorized into three main approaches: physical, chemical, and biological methods [194, 195].

Physical approaches utilize different forms of energy, such as light, electrical, or mechanical forces, to facilitate drug loading. Techniques frequently used in this category include electroporation, sonication, freeze–thaw cycles, and extrusion [194]. Electroporation, for example, involves the application of an electric field to a mixture of exosomes and therapeutic compounds, temporarily disrupting the bilayer membrane by creating pores that allow the drug to be introduced into the exosome [18]. This method has been successfully utilized to load miRNA, superparamagnetic iron oxide nanoparticles, bioactive macromolecules, as well as small‐molecule chemotherapeutic agents [194]. Sonication involves the use of a probe to transiently disrupt and reorganize the exosome lipid bilayer, enabling efficient drug loading [194]. The freeze–thaw method entails rapid cycles of freezing and thawing, which distort the lipid bilayer, thereby promoting drug encapsulation [194]. Extrusion forces exosomes through a porous membrane using a lipid extruder, disrupting the membrane to facilitate the penetration of therapeutic molecules [18, 194].

Chemical methods primarily involve saponin‐assisted permeabilization and the use of transfection reagents, which depend on membrane permeabilization and electrostatic interactions [194]. Saponin‐assisted permeabilization enhances the permeability of the exosome lipid bilayer without significantly disrupting its structural integrity [190]. Exosomes possess the natural capability to transport DNA, RNA, noncoding RNA, and miRNA, making them highly compatible with systems designed for nucleic acid delivery. For the purpose of encapsulation through transfection agents, various chemical substances are commonly employed, including calcium phosphate, polyethyleneimine, diethylaminoethyl‐dextran, and notably, liposomes [194].

Biological approaches, including incubation and viral transduction, are acknowledged for their exceptional loading efficiency and limited effects on the structure and function of exosomes [194]. Incubation is a simple technique where drug solutions are combined with exosome suspensions and incubated at room temperature, with factors such as drug size, charge, and hydrophobicity affecting loading efficiency [190]. Viral transduction introduces viruses into donor cells to overexpress or modulate specific genes, thereby loading genetic material and their expressed products into exosomes [194]. Each method presents unique advantages and limitations, offering diverse opportunities for the development of exosome‐based DDSs.

#### 2.5.2 Targeted Modification of Exosomes

Exosomes are essential for intercellular communication, making them particularly efficient as natural transporters of diverse bioactive molecules. Relative to other nanocarrier systems, exosomes possess reduced immunogenicity, enhanced biocompatibility, and an exceptional ability to traverse biological barriers, including the BBB [194, 196, 197, 198]. To optimize the targeting efficiency of exosomes, precise surface modifications are often implemented, exploiting their intrinsic tropism and cellular origin. Presently, modifications to exosome targeting are predominantly accomplished through genetic engineering, chemical alterations, and physical modification strategies. These approaches typically harness specific ligand–receptor interactions to enhance exosome binding to target cells, thereby facilitating endocytosis [199, 200, 201].

Genetic engineering techniques entail the fusion of a gene sequence encoding a targeting protein or peptide with exosomal membrane protein genes, followed by expression in donor cells [202, 203]. This process enables the engineered exosomes to present targeting ligands on their surface. For instance, the desired ligands can be expressed by transfecting cells with their corresponding coding sequences through viral vectors, resulting in exosome presentation of specific peptides [204, 205]. Numerous patents concerning exosomal surface modifications have been registered, including a notable one detailing the creation of therapeutic exosomes enriched with specific proteins in the exosomal lumen, such as brain acid soluble protein 1 or its fragments [206]. These techniques exploit membrane proteins to generate therapeutic exosomes by incorporating proteins that have been newly identified as being enriched on the exosome surface [207]. For example, Kim et al. [208] successfully developed T7 peptide‐modified exosomes that showed efficient targeting and delivery to C6 glioma cells. This research group further engineered curcumin‐loaded exosomes (Exo‐Cur) conjugated to retinol binding protein, significantly improving the intracellular delivery efficiency of curcumin [194]. Collectively, these studies highlight the immense potential of engineered exosomes in advancing targeted DDSs. The research group headed by Li et al. [209] successfully engineered exosomes for the transport of RNA by fusing CD9 with HuR. Bai et al. introduced an innovative tLyp‐1‐functionalized exosome by transfecting HEK293T cells with the tLyp‐1‐light2b gene [209, 210]. Additionally, Bliss and colleagues [211] attached silver particles (Ag) to the C1C2 domain of lactadherin, creating an exosome intended to mitigate immune responses to human adenovirus serotype 5. This exosome also enhanced the immunogenicity of both adenovirus serotype 5 and ChAdOx1 vaccines. In another investigation, Shi et al. [212] developed multivalent antibody‐retargeted exosomes (SMARTExos) by incorporating the human PDGFR transmembrane domain. These engineered exosomes exhibited functionalized CD3 and HER2 monoclonal antibodies on their surfaces, demonstrating significant and highly specific anticancer effects in both in vitro and in vivo settings [212].

Chemical modification techniques involve covalent or noncovalent attachment of proteins, peptides, aptamers, lipids, or polymers to exosomes. Frequently employed methods include click chemistry, azide‐alkyne cycloaddition, as well as various noncovalent techniques [213]. Tian et al. [166] covalently functionalized the surface of MSC‐derived exosomes with the cyclic peptide [c(RGDyK)], enhancing their targeting efficiency. In a similar vein, Jia et al. [214] and Kim et al. [215] reported improved targeting efficiency in exosomes that were modified using covalent approaches. Furthermore, several noncovalent strategies, including receptor–ligand interactions, electrostatic forces, and hydrophobic effects, have been utilized [213]. Qi et al. [216] employed receptor–ligand binding to affix superparamagnetic magnetite colloidal nanocrystal clusters to the membrane surface of reticulocyte‐derived exosomes, successfully targeting the TFR.

Similarly, Kim et al. [208], Choi et al. [217], and Dusoswa et al. [218] employed this approach to engineer modified exosomes. Nakase and Futaki [219] utilized electrostatic interactions to attach cationic lipids and the pH‐sensitive fusogenic peptide to negatively charged exosomes sourced from HeLa cells. Additionally, Tamura et al. [220] applied electrostatic interactions to enhance exosomes by incorporating cationized pullulan. Vandergriff et al. [221] harnessed hydrophobic interactions to specifically direct cardiac tissue targeting by linking heart‐homing peptides (such as CHP and CSTSMLKAC) to cardiac stem cell‐derived exosomes using DOPE‐NHS linkers, resulting in improved accumulation of exosomes in the heart. Similarly, Wang et al. [222] developed RGD‐engineered exosomes through hydrophobic interaction techniques. Sato et al. [223] fused exosomes with liposomes and utilized a freeze–thaw cycle in liquid nitrogen to create exosome–liposome hybrids, which exhibited superior fusion efficiency with HeLa cells compared with exosomes derived from RAW 264.7 macrophages or HeLa cells. Concurrently, Lee et al. [224] incorporated hybrid strategies that included fusogenic lipid liposomes to modify exosomes.

Physical modification techniques often encompass the incorporation of physical mediators, such as magnetic nanoparticles, into exosomes. For example, Jia et al. [214] developed an exosome system that integrates superparamagnetic iron oxide nanoparticles with curcumin, which, when combined with targeting peptides, facilitates precise imaging and therapeutic intervention for glioblastoma. In a similar study, Zhuang et al. [225] generated exosomes loaded with BAY55‐9837 in conjunction with superparamagnetic iron oxide nanoparticles. When subjected to an external magnetic field, these exosomes significantly prolonged drug half‐life and enhanced pancreatic targeting, thereby improving glycemic response in type 2 diabetes.

Notwithstanding these advancements in exosome engineering technologies, several challenges persist that hinder their clinical application. First, to mitigate exosome damage or aggregation under inappropriate temperature, pressure, or osmotic conditions, the functionalization process must be meticulously controlled [225]. Furthermore, the introduction of targeting components may jeopardize the multifunctional capabilities of exosomes [226, 227]. Additionally, the selection of suitable purification methods and the optimization of yields are essential for effectively isolating modified exosomes. They are equally important for excluding unmodified counterparts [228, 229]. Clinically, the production and concentration of sufficient quantities of exosomes pose significant challenges, as studies and clinical trials demonstrate that exosome efficacy is profoundly influenced by their protein cargo, quantity, and size [229]. Moreover, the variability in cell sources, therapeutic dosages, delivery routes, and timing further constrains the therapeutic potential of exosomes [230].

## 3 The BBB Permeability of Exosomes

**FIGURE 5:**
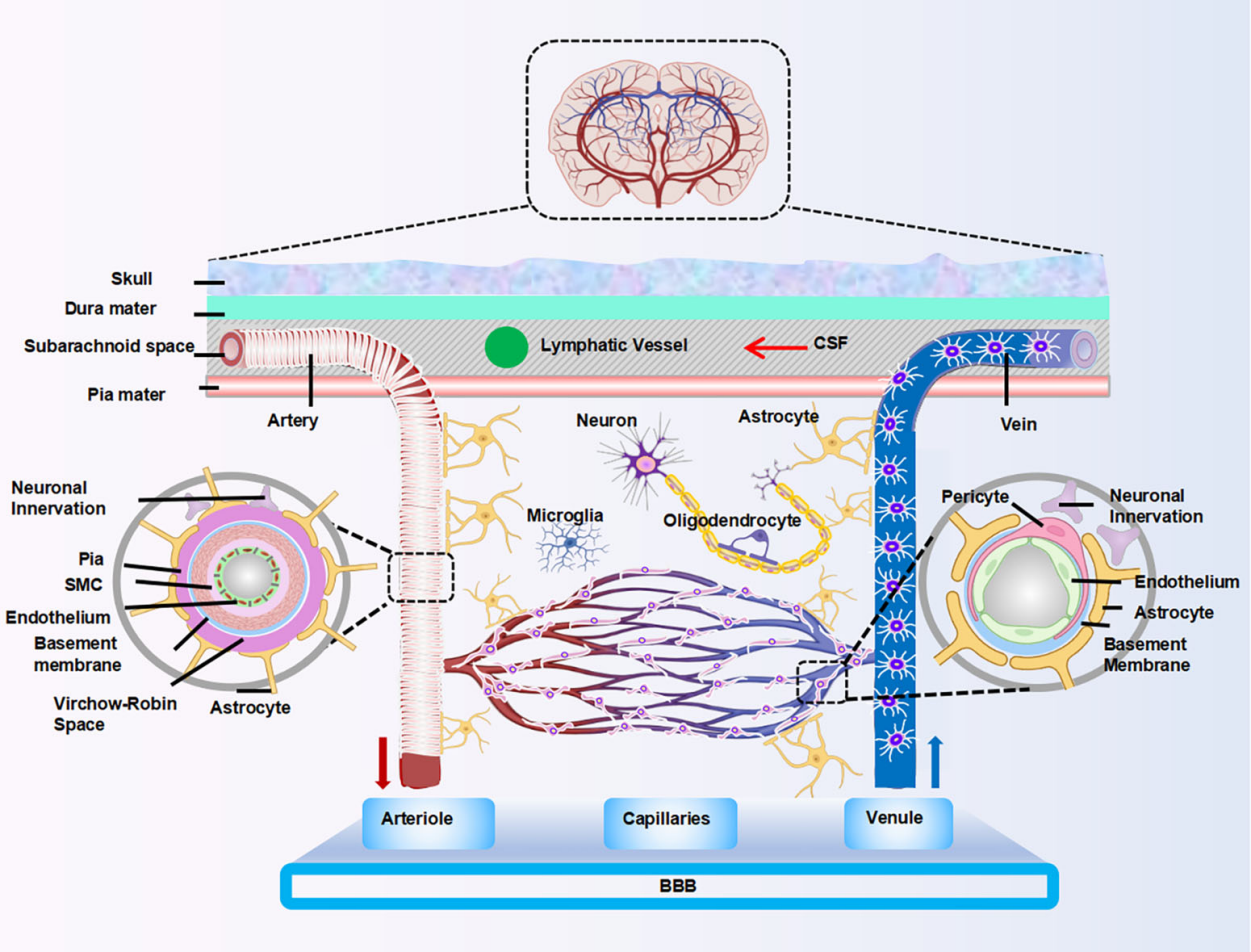
The NVU. The NVU consists of various cell types, including ECs and mural cells (pericytes) along brain capillaries, venules, and arterioles, as well as SMCs in arterioles, small arteries, and veins. Glial cells (astrocytes, microglia, oligodendrocytes) and neurons are also key components. Endothelial and mural cells exhibit molecular differences across vascular segments, resulting in arterio‐capillary‐venous zonation. In penetrating arteries (left inset), ECs are surrounded by 1–3 layers of SMCs and the pia mater, with the Virchow‐Robin space separating the pia from the glia limitans. As vessels transition to arterioles, the SMC layer becomes a single layer, and ECs remain continuous with those in capillaries. At the capillary level (right inset), ECs and pericytes share a basement membrane and form TJs, while astrocytic endfeet envelop the vessel walls. Neuronal connections are observed in SMCs, pericytes, and astrocytes. The BBB, a key part of the NVU, consists of tightly sealed ECs that limit permeability, particularly at brain capillaries. BBB, blood–brain barrier; CSF, cerebrospinal fluid; ECs, endothelial cells; NVU, neurovascular unit; SMCs, smooth muscle cells; TJs, tight junctions.

The CNS has established multiple protective barriers, each with unique permeability properties, to safeguard the brain from pathogens, neurotoxic agents, and elements present in the bloodstream. Among these barriers are the blood–cerebrospinal fluid (CSF) barrier, the BBB, the blood–retinal barrier, and the blood–spinal cord barrier. Notably, the BBB is the most complex of these structures, playing a vital role within the neurovascular unit (NVU) by enabling interactions between ECs and various types of neural and immune cells. The NVU is composed of multiple specialized cell populations, encompassing vascular components (ECs, pericytes, and vascular smooth muscle cells [SMCs]), glial elements (astrocytes and microglia), and neuronal cells (Figure 5). Its central function lies in the regulation of cerebral blood flow, facilitating rapid alterations in blood velocity and optimizing oxygen delivery to targeted brain regions when needed [231]. The BBB is chiefly constituted by tightly joined brain microvascular ECs (BMECs), with critical support from astrocytes, pericytes, and other neighboring cells. It is crucial in maintaining both the structural integrity and functional efficiency of the NVU [232]. This section will examine the structural and physiological properties of the BBB.

### 3.1 Structural Components of the BBB

The BBB demonstrates highly selective permeability, rigorously controlling molecular flux to safeguard the brain from potentially harmful substances and pathogens. As depicted in Figure 5, the structural integrity of the BBB is preserved through complex interactions among BMECs, astrocytes, pericytes, and other supporting cells, collectively stabilizing the NVU. Nevertheless, this stringent selectivity presents significant challenges for drug delivery, as the presence of efflux transporters frequently reduces drug concentrations within the brain, thus limiting therapeutic efficacy. Consequently, a comprehensive understanding of the structural and functional properties of the BBB is crucial for formulating effective therapeutic strategies for addressing brain‐related disorders.

#### 3.1.1 Brain Microvascular ECs

BMECs represent the fundamental structural component of the BBB and exhibit distinct structural and functional characteristics relative to peripheral ECs [233, 234, 235]. Morphologically, BMECs demonstrate an elevated mitochondrial density to support the substantial metabolic requirements of their active transport mechanisms [236, 237]. In contrast to peripheral ECs, BMECs completely lack fenestrations, specialized transcellular pores that facilitate molecular exchange. This structural distinction significantly limits paracellular permeability and enhances the barrier properties of the BBB. Peripheral ECs, by comparison, typically exhibit abundant fenestration structures that enable enhanced vascular permeability and more extensive molecular trafficking.

BMECs are linked by TJs and adherens junctions. These junctions establish a distinct luminal–abluminal membrane structure that is characteristic of the BBB [235]. These cells express TJ proteins (TJPs), including claudins, occludins, and zonula occludens (ZO) proteins, which effectively prevent the passage of most molecules, except small lipid‐soluble compounds [238]. Additionally, BMECs harbor efflux transporters such as P‐gp and breast cancer resistance protein, which play active roles in removing harmful compounds and pharmaceuticals, thereby facilitating the occurrence of multidrug resistance. Furthermore, BMECs employ receptor‐mediated transport (RMT) mechanisms to facilitate the absorption of vital nutrients, including glucose, amino acids, purines, and nucleosides, from the circulation into the brain [239, 240].

A notable feature of BMECs is their overall negative surface charge, which serves to repel negatively charged molecules and reduce the expression of leukocyte adhesion molecules. This mechanism effectively limits the infiltration of immune cells into the brain [241, 242]. BMECs also exhibit high transepithelial electrical resistance, thereby minimizing transcellular vesicular transport across the blood vessel walls [243, 244].

Although BMECs constitute the physical foundation of the BBB, its functional integrity is heavily reliant on the interactions with other cell types. Astrocytes extend their endfeet to enwrap brain capillaries, whereas pericytes are embedded within a shared basement membrane with ECs. In addition, perivascular microglia and the basal lamina play a significant role in preserving the integrity of the BBB at the capillary and postcapillary venule levels [245, 246].

#### 3.1.2 SMCs and Brain Pericytes

SMCs serve as essential structural elements of vessel walls, widely distributed throughout internal organs, notably within the gastrointestinal and vascular systems, with a pronounced presence in the cerebrovascular network. Within the brain, SMCs within the brain interact closely with both endothelial and neural cells, contributing significantly to the regulation of cerebrovascular tone and the preservation of BBB integrity. Although traditionally regarded as indirect contributors to BBB formation, cerebrovascular SMCs collaborate with neural cells to regulate vasoconstriction and vasodilation, thereby indirectly influencing BBB permeability [247, 248, 249].

In contrast, pericytes are essential components of the NVU. Residing within the basement membrane of cerebral capillaries and positioned in close proximity to ECs, pericytes establish direct physical contacts and mediate paracrine signaling, playing pivotal roles in regulating BBB metabolism and maintaining its structural integrity [247, 250, 251]. Pericytes not only envelop ECs but also actively participate in the BBB's metabolic functions, including the facilitation of ion exchange. The shared continuous basement membrane between pericytes and ECs is fundamental to the formation of TJs, which are crucial for preserving BBB impermeability. Additionally, pericytes fortify the barrier's integrity by downregulating endothelial permeability‐related genes and upregulating TJPs such as claudin‐5, occludin, and ZO‐1 [249, 250]. Experimental studies have demonstrated that the depletion of pericytes significantly increases BBB permeability, highlighting their critical role in preserving the barrier's function [252]. Pericytes also regulate BBB permeability by releasing factors that modulate TJ dynamics in ECs and influence the polarization of astrocytic endfeet.

Beyond their regulatory functions, pericytes possess stem cell‐like characteristics, aiding in the repair and angiogenesis of cerebral blood vessels through “intercellular crosstalk” with other NVU components, promoting vascular stability and regeneration [252]. However, recent research has cast doubt on the contractile capacity of brain pericytes, suggesting that their function in neurovascular coupling may vary from that of SMCs, particularly in brain capillaries and arteries [253]. Neurovascular coupling, an essential process that modulates cerebral blood flow based on neuronal activity, demonstrates variability across different regions [254]. Despite this, SMCs continue to be the primary regulators of blood flow under both physiological and pathological conditions. Arteriolar SMCs, which form circumferential structures within the vessel walls, are essential for maintaining vascular tone [255]. Notably, small arteriolar SMCs in the human and mouse brain express α‐smooth muscle actin, a cytoskeletal protein integral to their contractile function. This expression highlights their pivotal role in maintaining vascular tone and regulating cerebral blood flow dynamics.

#### 3.1.3 Astrocytes

Astrocytes, the most prevalent glial cells in the CNS, display both structural distinctiveness and functional adaptability, playing a vital role in sustaining neural homeostasis [256]. Defined by their characteristic star‐shaped morphology and extensively branched processes, astrocytes offer critical structural support to neurons, closely enveloping cerebral microvascular ECs [257]. This intimate interaction establishes a cellular network integral to the integrity of the BBB, protecting brain from potentially harmful agents and maintaining distinct fluid environments [258].

Astrocytes exhibit exceptional functional adaptability. Astrocytes secrete pivotal signaling molecules such as sonic hedgehog, vascular endothelial growth factor, angiopoietin‐1, ACE‐1, and apolipoprotein E (ApoE), which provide essential metabolic support to adjacent ECs and stabilize the BBB by inducing specialized endothelial phenotypes that fortify its integrity [259]. Furthermore, astrocytes are involved in various processes within the CNS, such as providing support to neurons, regulating metabolism, maintaining potassium ion balance, facilitating dynamic intercellular communication, clearing metabolic waste, modulating cerebral blood flow, regulating vascular tone, and coordinating neuroimmune responses [260, 261, 262].

Although the exact role of astrocytes in BBB function remains a subject of ongoing debate, their indispensable presence at the neurovascular interface between neurons and ECs is widely recognized [263, 264]. The diverse structures and functions of astrocytes underscore their essential role in preserving neural function and ensuring the integrity of the BBB.

#### 3.1.4 Microglia

Microglia, frequently referred to as the resident “macrophages” [265] of the CNS, represent vital elements of the NVU and are integral to immune surveillance [266]. These cells emerge early in CNS development, prior to the migration of ECs into the brain, and actively participate in the regulation of cerebrovascular formation. Microglia, derived from hematopoietic progenitors, migrate into the CNS parenchyma to offer essential immune defense within the BBB. In response to inflammatory stimuli, microglia initiate both cellular and humoral immune responses, thereby triggering a cascade of protective mechanisms [267].

Microglia, despite comprising only approximately 10% of CNS cells, are extensively found throughout both gray and white matter, where they are essential for neuroinflammatory mechanisms. Upon activation, microglia undergo considerable morphological alterations and secrete various proinflammatory cytokines, including interleukin‐1 (IL‐1) and tumor necrosis factor‐alpha (TNF‐α), along with chemokines that enhance the inflammatory process [268]. These cells demonstrate notable plasticity, enabling transitions between proinflammatory (M1) and anti‐inflammatory or repair‐promoting (M2) states. However, emerging studies suggest that the differences in phenotypes may be more complex than previously understood [269].

It is essential to distinguish microglia from perivascular macrophages within the BBB. While perivascular macrophages, which originate from the monocyte lineage, primarily participate in phagocytic clearance and BBB extravasation, microglia derive from the yolk sac during early embryonic development [270]. In addition to their role in innate immunity as antigen‐presenting cells, microglia are also involved in neuronal development [265]. Notably, selective depletion of microglia leads to a significant reduction in blood vessel density in mice, underscoring their critical role in maintaining CNS homeostasis during steady‐state conditions [271].

#### 3.1.5 Neurons

Neurons serve as the fundamental structural and functional units of the CNS, playing a pivotal role in the reception, integration, and processing of information, which facilitates the establishment of complex neural networks. While neurons are not directly implicated in the physical construction of the BBB, they significantly affect its integrity and functionality through their intimate spatial relationships with the barrier structures formed by capillary ECs and their TJs, typically maintaining a distance not exceeding 25 µm.

This intimate proximity renders neurons exceedingly reliant on the stability of the microenvironment upheld by the BBB, especially concerning ionic balance and nutrient availability [272]. Furthermore, neurons indirectly regulate both blood flow and BBB permeability by modulating the stability of TJs and influencing the activity of efflux transporters. This regulatory capacity is essential for neurons to effectively perform their physiological functions within a stable and nutrient‐rich environment [273].

#### 3.1.6 Basement Membrane

The basement membrane represents a crucial component of the BBB, modulating interactions between cells and the extracellular matrix, thereby influencing the morphology and functionality of the barrier [274]. In the intricate structure of the BBB, the basement membrane is classified into two main types: the endothelial basement membrane and the parenchymal basement membrane. Each type serves distinct and specific functions. The endothelial basement membrane, which is intimately associated with capillary ECs, predominantly consists of collagen IV, laminin, and fibronectin. This composition not only provides critical structural support but also enhances the integrity of the barrier. In contrast, the parenchymal basement membrane is more complex, comprising various extracellular matrix components, in addition to laminin, astrocytes, and their end‐foot processes, thus establishing a protective barrier within brain tissue [275, 276].

Both types of basement membranes are indispensable; the endothelial basement membrane is positioned between ECs and astrocytes, whereas the parenchymal basement membrane extends further into the brain. Collectively, these membranes provide essential structural support and mechanical stability to the BBB. Additionally, they actively participate in regulating substance transport, signal transduction, and other critical physiological processes. The continuity and integrity of these basement membranes are vital for sustaining BBB function, serving as the first line of defense against harmful substances that seek to infiltrate brain tissue [277, 278]. Injury to these membranes may interfere with protein expression in ECs, resulting in heightened permeability that may facilitate the infiltration of inflammatory cells, including leukocytes, into the CNS under pathological circumstances, ultimately undermining its standard physiological condition.

### 3.2 BBB Alteration in Brain Diseases

As previously articulated, the BBB constitutes a highly specialized structure composed of brain ECs, astrocytes, pericytes, and the basement membrane, along with various additional cellular constituents. This complex physiological barrier fulfills several critical functions: (1) preventing the ingress of pathogens, including bacteria, viruses, neurotoxic substances, inflammatory mediators, immune cells, and other detrimental agents into the brain, thereby safeguarding neural tissue from injury; (2) ensuring an adequate blood supply to support vital physiological processes, including sensory nerve conduction and temperature regulation; (3) regulating cerebral hemodynamics, such as cerebral blood flow and intracranial pressure, to facilitate normal brain metabolism and function; and (4) facilitating the selective transport of essential nutrients, including glucose and amino acids, to promote brain development and maintain homeostasis.

**FIGURE 6:**
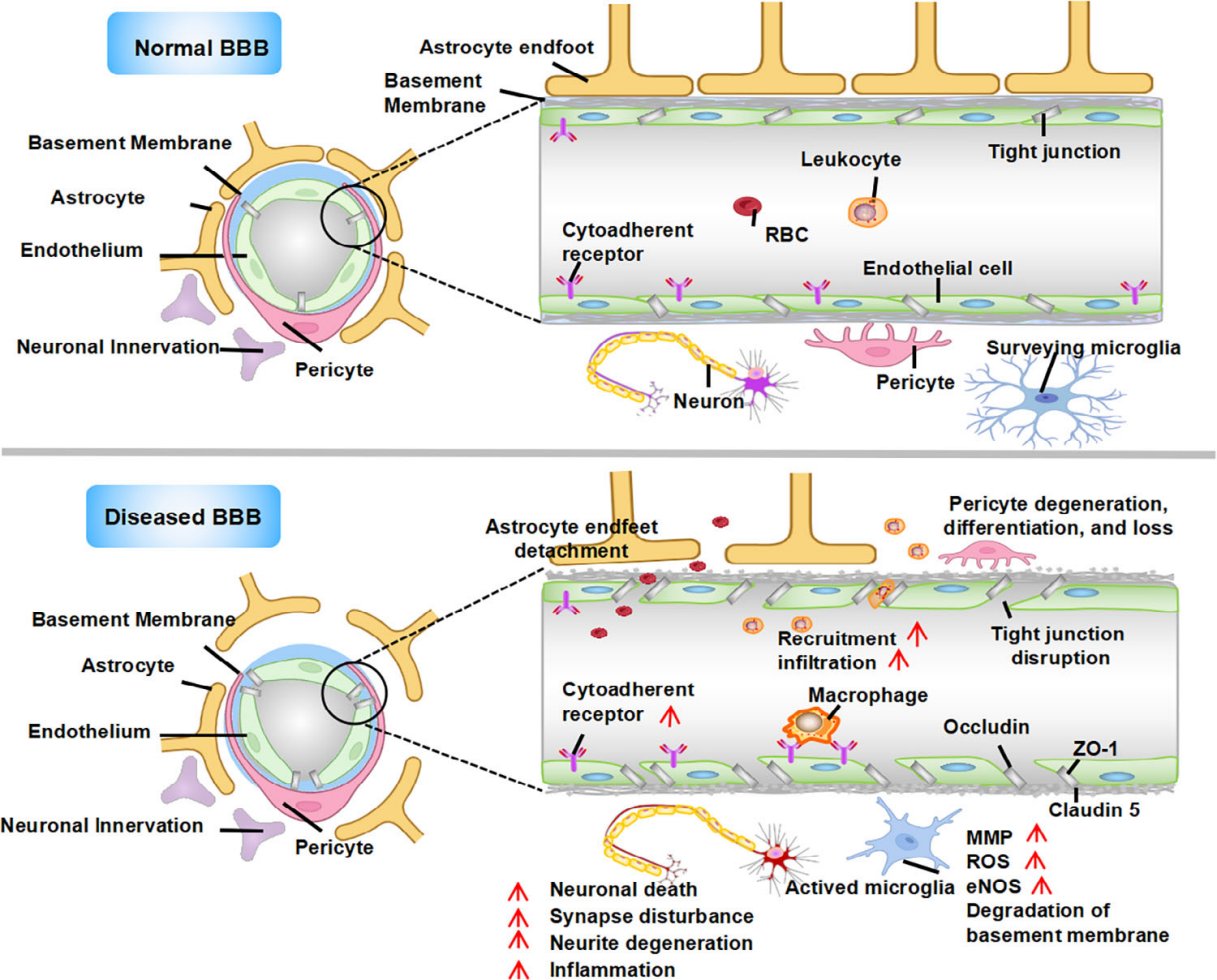
The composition of the BBB and key alterations in pathological conditions. Under physiological conditions (upper inset), the BBB is formed by vascular ECs connected by adherens junctions and TJs. These ECs are supported by pericytes, a basement membrane, and astrocytic end‐feet. Neurons and microglia contribute to the BBB's structural and functional integrity. In pathological conditions (lower inset), the BBB becomes more permeable due to increased activity of MMPs, elevated ROS and NO levels (produced by eNOS in ECs and iNOS in activated microglia/macrophages). Activated microglia and macrophages release cytokines and chemokines, which degrade the basement membrane and disrupt tight junction proteins, such as occludin, ZO‐1, and claudin‐5. These molecular changes initiate inflammation, leukocyte recruitment, brain infiltration, neuronal dysfunction, and neurodegeneration. BBB, blood–brain barrier; CSF, cerebrospinal fluid; ECs, endothelial cells; eNOS, endothelial nitric oxide synthase; iNOS, inducible nitric oxide synthase; MMPs, matrix metalloproteinases; NO, nitric oxide; RBC, red blood cell; ROS, reactive oxygen species; SMCs, smooth muscle cells; TJs, tight junctions; ZO‐1, zonula occludens‐1.

In physiological states, the BBB employs various mechanisms, including TJs, active efflux systems, and specialized transporters, to maintain its selective permeability. This regulation is vital for ensuring proper brain function and overall health. Conversely, under pathological conditions, both the integrity and function of the BBB may be disrupted, resulting in altered permeability and changes in transporter expression. Increased permeability of the BBB has been noted in several neurological disorders, allowing detrimental substances and inflammatory agents to penetrate the brain, consequently exacerbating pathological damage (Figure 6).

### 3.3 Mechanism of Exosome Transport Across the BBB

**FIGURE 7:**
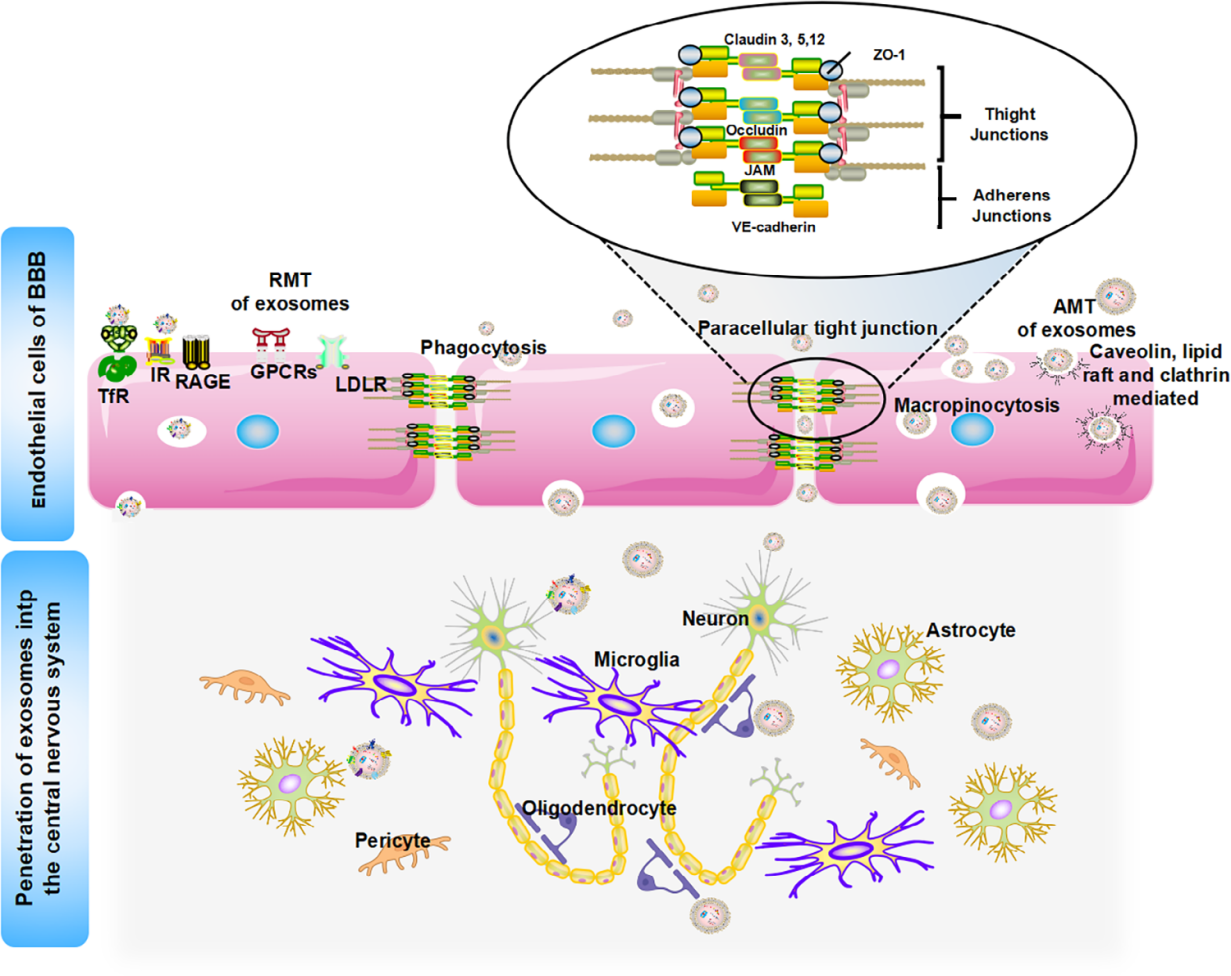
Structure of the BBB and mechanisms of exosome crossing. The BBB is a highly specialized structure composed of ECs, pericytes, astrocytes, oligodendrocytes, microglia, and neuronal terminations. Its selective permeability is attributed to TJs and AJs between ECs, which restrict paracellular transport. Critical proteins involved in these junctions include claudin‐3, claudin‐5, claudin‐12, occludin, JAMs, ZO‐1, and VE‐cadherin. Exosomes, small vesicles that mediate intercellular communication, can traverse the BBB via several pathways. RMT involves the engagement of specific receptors such as TFR, IR, LDLR, GPCRs, and RAGE. Beyond RMT, exosomes can cross biological barriers via alternative mechanisms. These include: (1) lipid raft‐associated nonselective endocytosis and (2) macropinocytosis, both of which may be classified as subtypes of, or potentially augmented by AMT. Furthermore, exosomes may utilize paracellular transport by modulating tight junction integrity, or directly attach to the plasma membrane, followed by internalization and subsequent release of their vesicular contents into the cytoplasm. AJs, adherens junctions; AMT, adsorptive‐mediated transcytosis; BBB, blood–brain barrier; CMT, carrier‐mediated transport; ECs, endothelial cells; GPCRs, G protein‐coupled receptors; IR, insulin receptor; JAMs, junctional adhesion molecules; LDLR, low‐density lipoprotein receptor; RAGE, receptor for advanced glycation end‐products; RMT, receptor‐mediated transport; TFR, transferrin receptor; TJs, tight junctions; VE‐cadherin, vascular endothelial cadherin; ZO‐1, zonula occludens‐1.

Exosomes cross the BBB by leveraging their unique biological properties and the specialized structure of the barrier's ECs. Multiple mechanisms work together to facilitate this transport. Although the mechanisms underlying exosome traversal of the BBB are not yet fully elucidated [279], studies indicate that exosomes achieve targeted delivery through multiple pathways, primarily including: RMT; nonselective endocytosis associated with lipid raft domains and macropinocytosis—both of which may fall under or be enhanced by AMT; paracellular transport related to TJs; and exosome attachment to the plasma membrane followed by internalization, releasing vesicular contents into the cytoplasm (Figure 7) [279, 280, 281, 282].

RMT plays a critical role in enabling exosomes to cross the BBB. Surface ligands on exosomes, such as integrins, tetraspanins, and heat shock proteins, selectively bind to specific receptors on brain ECs and initiate internalization. Among these receptors, the TFR is the most widely studied. Studies have shown that exosomes functionalized with TFR‐binding peptides exhibit significantly enhanced BBB permeability and improved delivery of therapeutic agents to the CNS [283, 284]. In addition to TFR, several other receptors have been identified as potential mediators of exosome transcytosis across the BBB. These include the low‐density lipoprotein receptor, insulin receptor, G‐protein‐coupled receptors (GPCRs), and receptor for advanced glycation end‐products (RAGE) [285]. Modifying exosomes with ligands that target these receptors offers a promising approach to further enhance delivery efficiency to the CNS [279]. Simultaneously, nanoparticle‐based functionalization of exosomes has been applied to improve targeting accuracy, minimize off‐target interactions, and increase accumulation in brain tissues. Importantly, the efficiency of RMT is influenced by the affinity between ligand and receptor, as well as the receptor's recycling capability. Therefore, selecting appropriate receptor–ligand combinations is essential for achieving precise and effective therapeutic outcomes [286].

To cross the BBB effectively, exosomes must first recognize and bind to BMECs. Among various transport pathways, membrane fusion offers distinct advantages. It allows exosomes to bypass the conventional endocytosis–endosome–lysosome route, thereby avoiding intracellular cargo degradation. Instead, it facilitates the direct cytoplasmic release of therapeutic biomolecules such as proteins, RNAs, and lipids into target cells. This fusion relies on the direct merging of exosomal and endothelial membranes, regulated by lipid components including PS, cholesterol, and sphingomyelin. Lipid raft microdomains, in particular, promote membrane curvature and enhance fusogenic activity, ultimately improving fusion efficiency [287]. The efficiency of membrane fusion is strongly influenced by membrane lipid composition. PS binds specifically to receptors such as T‐cell immunoglobulin and mucin domain‐containing 4 and lactadherin, facilitating membrane docking and fusion [288]. Cholesterol increases membrane fluidity and plasticity, aiding the fusion process [289], while sphingomyelin contributes to rigidity, stabilizing fusion sites and preventing premature cargo leakage or degradation [290]. Fusion efficiency also varies across exosome subtypes and tissue origins, reflecting differences in lipid composition and surface protein expression. For example, neuron‐derived exosomes are rich in neural adhesion molecules, which improve their targeting and fusion with brain ECs [291]. Lipidomic modifications such as increasing PS levels can further boost exosomal fusogenic potential, making them more effective drug delivery vehicles [292]. In addition, fusion‐related proteins like the SNARE complex and Rab GTPases play crucial roles in mediating membrane docking and fusion, ensuring efficient cytosolic delivery of exosomal cargo [293]. Recent studies also demonstrate that modulating lipid raft dynamics or incorporating synthetic fusogenic peptides can significantly enhance fusion efficiency, advancing the therapeutic use of exosomes in CNS disorders [294].

In addition to vesicle‐related pathways such as RMT and AMT, carrier‐mediated transport (CMT) is also an important mechanism for substance transport across the BBB. CMT is a highly selective transport process primarily responsible for moving small hydrophilic molecules such as glucose, amino acids, and vitamins from the bloodstream into brain tissue, thereby meeting the stringent metabolic demands of the brain. This process relies on specific membrane transporters located on the apical (luminal) and basolateral (abluminal) membranes of BMECs [295, 296].

For example, glucose transporter 1 is abundantly expressed at the BBB and efficiently facilitates glucose uptake into the brain [297, 298]. Similarly, large neutral amino acid transporter 1 transports essential amino acids such as leucine, tyrosine, and phenylalanine, and has also been shown to mediate the transport of certain drug precursors or substrate mimetics [299, 300]. However, unlike RMT, which depends on specific receptor–ligand recognition and can mediate transcytosis of nanoscale carriers like exosomes via endocytic vesicle formation, CMT is structurally restrictive. It is limited to small molecules that closely resemble endogenous substrates in structure. Given that exosomes are vesicular structures approximately 30–150 nm in diameter, significantly larger than substrates typically transported by CMT, and lack molecular features recognized by carrier proteins, they cannot serve as substrates for CMT across the BBB.

Therefore, utilizing the RMT and AMT pathway is a common approach for transporting therapeutics across the BBB [213]. For instance, studies have revealed that engineered exosomes can be tailored to target brain cells via specific ligands on their membranes, facilitating effective penetration into brain tissue through receptor‐mediated transcytosis. These exosomes transport therapeutic molecules, including miRNAs (e.g., miR‐124, miR‐21) and anti‐inflammatory agents (e.g., IL‐10), capable of mitigating neuroinflammation induced by Aβ or tau proteins. The primary advantages of this approach include the reduction of peripheral side effects and a significant enhancement in drug‐targeting efficiency, thereby providing a more precise therapeutic strategy [301, 302].

## 4 Exosome‐Mediated BBB Crossing: Biological Mechanisms and Targeted Modifications

Extensive research demonstrates that exosomes, due to their unique role in intercellular communication and delivery of bioactive molecules, can efficiently cross the BBB. The BBB, mainly composed of ECs connected by TJs, strictly regulates molecular entry into the CNS to maintain cerebral homeostasis (Figure 5) [227, 303]. As discussed in Section 2.3, although the exact mechanisms of exosomal BBB crossing remain unclear, key pathways include receptor‐mediated transcytosis, lipid raft‐dependent endocytosis, macropinocytosis, paracellular transport through TJs, direct fusion with the plasma membrane for cargo release, and activation of intracellular signaling via GPCRs (Figure 7) [296, 304]. Importantly, exosomal surface proteins, such as integrins, tetraspanins, and lipids play critical roles in modulating BBB permeability [305]. Additionally, exosomes deliver nucleic acids, peptides, and proteins to ECs, facilitating their transit across the BBB [306]. Genetic engineering and chemical modifications have further enhanced the brain‐targeting abilities of exosomes [19]. Moreover, different administration routes and pathological states also improve exosomal penetration into brain tissue [307].

The following discussion will further delve into the fundamental mechanisms by which exosomes traverse the BBB, with a particular focus on their surface marker composition and precise molecular targeting strategies to enhance the efficacy and potential of brain‐directed delivery.

### 4.1 Exterior Constituents of Exosomes Facilitate Brain Permeability

In brain‐targeted delivery systems, the membrane components of exosomes, including integrins, tetraspanins, and specific lipids, serve as key facilitators of transport across the BBB. For instance, exosomes originating from macrophages can improve BBB permeability by interacting specifically with C‐type lectin receptors on BMECs through membrane‐bound integrin LFA‐1 [308, 309]. Tetraspanins, which are among the most abundant protein families present on the exosomal surface, contribute significantly to the regulation of interactions between exosomes and ECs and support their transendothelial migration [310]. Moreover, the lipid composition of exosomal membranes plays a crucial role in determining their capacity to penetrate the BBB [311, 312]. In a three‐dimensional (3D) static BBB model, Jakubec and colleagues [305] found that exosomes rich in lysophospholipids, as opposed to those containing PS, were more readily internalized by ECs and successfully crossed the barrier. Collectively, these observations emphasize the vital function of inherent exosomal surface molecules in enabling BBB traversal and underscore their structural and functional importance in brain‐targeted drug delivery.

### 4.2 Influence of Exosomal Origin on Brain Targeting

The origin of exosomes, whether at the tissue or cellular level, plays a pivotal role in directing their organotropism, particularly in the context of brain‐specific delivery. Accumulating evidence has underscored that the phenotype of the donor cell has a profound influence on the biodistribution and in vivo localization of exosomes. For instance, Alvarez‐Erviti et al. [313] demonstrated that exosomes engineered from specific cell types are capable of traversing the BBB to deliver small interfering RNA (siRNA) into neurons, thereby illustrating their therapeutic utility for CNS disorders. Correspondingly, work by Joshi and Zuhorn [307] indicated that exosomes secreted by NSCs exhibit a markedly enhanced capacity for CNS delivery relative to those derived from MSCs, as evidenced in murine models. In particular, exosomes from the immortalized NSC‐like C17.2 line have been shown to mediate the delivery of protein therapeutics across a human in vitro BBB model composed of BMECs, likely via endocytic uptake. This process appears to involve heparan sulfate proteoglycans (HSPGs), although it remains unresolved whether HSPGs function as active internalization receptors or serve solely as binding sites. Furthermore, Qu et al. [170] revealed that exosomes derived from blood cells are capable of BBB translocation through interactions with TFRs, enabling the delivery of dopamine and suggesting potential for therapeutic application in PD. Additionally, Grapp et al. [314] identified that exosomes secreted by epithelial cells of the choroid plexus can permeate the brain parenchyma and are selectively internalized by neuronal and astrocytic populations, thereby offering a pathway for targeted delivery within the CNS. Collectively, these findings emphasize that the cellular provenance of exosomes is a critical determinant of their ability to cross the BBB and target specific CNS cell types, which must be strategically considered in the design of brain‐targeted delivery systems. Nonetheless, despite these insights, the molecular mechanisms underpinning exosomal organotropism remain incompletely characterized, necessitating further systematic investigation to elucidate their regulatory pathways.

### 4.3 Targeting the Brain: Enhancing Exosome Specificity

Drug delivery to the CNS has long been limited by the highly‐selective BBB, which significantly restricts the penetration of therapeutic molecules. Recently, exosomes have emerged as promising carriers for treating neurological diseases due to their excellent biocompatibility and ability to cross the BBB. To improve their brain‐targeting specificity, various strategies have been proposed, including surface modifications to enhance recognition, leveraging the natural targeting abilities of parent cells, and designing hybrid systems that combine natural and artificial components for more efficient delivery.

Surface functionalization of EVs entails the modification of their membranes by conjugating specific ligands or molecules to enhance selective binding to targets within the CNS. For instance, transferrin ligands mediate the transcytosis of EVs across the BBB via interaction with TFRs. Studies have demonstrated that EVs modified with transferrin ligands can efficiently deliver chemotherapeutic agents to neural cells, significantly improving therapeutic outcomes in CNS tumors [315]. Similarly, EVs functionalized with neural cell adhesion molecule 1 ligands exhibit improved targeting specificity, effectively inhibiting tumor growth in glioblastoma models [214]. Additionally, Tian et al. [166] reported that curcumin‐loaded EVs, following surface modification, exhibit enhanced anti‐inflammatory and neuroprotective effects in ischemic stroke models, accompanied by a significant reduction in infarct volume.

Exosomes derived from specific CNS cell types, such as astrocytes and brain ECs, inherently exhibit brain‐targeting capabilities. Cao et al. [316] demonstrated that EVs originating from brain ECs can effectively transport mitochondria‐targeting photosensitizers across the BBB, thereby enhancing photodynamic therapy efficacy and selectively inducing apoptosis in glioblastoma cells. Furthermore, astrocyte‐derived exosomes can deliver genetic materials, including siRNA, to neurons, highlighting their significant potential in treating neurodegenerative disorders such as AD and PD [317, 318, 319].

To enhance the functionality and targeting efficiency of therapeutic carriers, researchers are investigating hybrid systems that combine exosomes with synthetic nanoparticles or liposomes. Khongkow et al. [320] developed a nanoplatform by integrating gold nanoparticles into exosomes, which significantly improved their ability to cross the BBB and enhanced brain‐targeting efficiency in a ND model. Similarly, the fusion of liposomes with exosomes has been shown to improve membrane stability and delivery precision. Liu et al. [321] constructed a hybrid exosome–liposome nanosystem labeled with a near‐infrared‐II fluorescent dye. This system exhibited superior light‐harvesting capability, a photothermal conversion efficiency of 62.28%, and achieved effective tumor ablation in a glioblastoma photothermal therapy model [321].

### 4.4 Alternative Approaches for Enhancing Exosomal BBB Penetration

Leveraging strategies to enhance exosome‐mediated brain delivery, researchers have investigated alternative routes such as intranasal (IN) administration to facilitate BBB penetration [322, 323]. IN delivery of catalase‐loaded exosomes achieves accumulation in the CNS and demonstrate neuroprotection in PD models [324]. Likewise, Zhuang et al. [325] found that murine lymphoma‐derived exosomes rapidly reach the brain via IN administration. Despite promising outcomes, the transport mechanisms remain unclear. Notably, pathological conditions, particularly neuroinflammation, have been shown to enhance exosome BBB crossing. For example, inflammation increases macrophage‐derived exosome brain entry threefold [308], and TNF‐α‐induced stroke models show enhanced exosome uptake and transport [326]. These findings indicate that disease states can be harnessed to optimize exosome‐based brain delivery, warranting further investigation.

## 5 Roles of Exosomes and Exosome‐Delivered Drugs in Different Types of NDs

Exosome‐based DDSs represent a promising strategy for treating CNS disorders, including NDs, brain tumors, and psychiatric conditions. Their biocompatibility and unique capacity to cross the BBB make them particularly well suited for precision neurology. NDs, such as AD, PD, HD, ALS, and MS, remain difficult to treat due to progressive neuronal loss and the BBB's restrictive properties. Conventional drug delivery approaches often fall short, either failing to achieve sufficient CNS penetration or causing off‐target effects. Exosome‐based therapies address these limitations by exploiting exosomes’ natural affinity for neural tissues, enabling targeted delivery of therapeutic agents across the BBB. This strategy not only enhances drug bioavailability but also offers the potential to alter disease progression by targeting the molecular mechanisms underlying neurodegeneration, rather than merely relieving symptoms. Tables 4, 5, and 6 respectively provide an overview of cell‐derived exosomes and their therapeutic applications in NDs.

**TABLE 4:** Overview of cell‐derived exosomes and their therapeutic applications in AD.

Source of exosomes	Cargo	Administration route	Key findings	Outcome	References
Curcumin‐treated (primed) cells	Cur	i.n.	Improved curcumin delivery by boosting solubility and targeting BBB transport through LFA‐1/ICAM‐1	Activated AKT/GSK‐3β to stop Tau phosphorylation, prevented neuron death, and eased AD symptoms	[173]
Macrophage‐derived exosomes	Sil	i.v.	Prevents Aβ aggregation and reduces neuroinflammation by inhibiting astrocyte activation	Improved cognitive function and alleviated cognitive impairment in AD mice	[161]
Plasma‐derived exosomes	Que	i.p.	Improved brain delivery of Que, reduced Tau phosphorylation, and lowered NFT formation	Significantly improved cognitive function and alleviated AD‐like symptoms in OA‐induced AD mice	[327]
Adipose‐derived stem cells	Coenzyme Q10	i.p.	Exo+CoQ10 improved memory, boosted BDNF and SOX2, and increased cell density.	Improved memory and cognition by increasing BDNF and SOX2	[328]
Human adipose tissue‐derived MSCs	Enzymatically active NEP	In vitro	ADSC‐exosomes with active NEP were absorbed by N2a cells, reduced Aβ levels, and showed higher NEP activity than BMSC‐exosomes.	Delivered active NEP and reducing Aβ levels	[329]
Dental pulp stem cells	Neurotrophic factors and cytokines	In vitro	DPSC secretome had stronger neuroprotective effects, reduced Aβ toxicity, increased Bcl‐2, decreased Bax, and degraded Aβ within 12 h.	Promoted cell survival and degrading Aβ peptide	[330]
Bone marrow MSCs	Not specified as a single cargo	Lateral ventricle injection	BMSC‐exos injected into the lateral ventricle improved AD‐like behavior, reduced inflammation, Aβ1‐42, and p‐Tau, inhibited glial activation, and increased BDNF and synaptic proteins.	Improved cognition and behavior in AD mice by reducing inflammation, lowering AD proteins, and boosting BDNF	[331]
MSCs	Various MSC‐derived bioactive factors	i.v.	MSC‐exosomes reduced Aβ, restored memory‐related genes, improved glucose metabolism and cognition, and regulated neurons and astrocytes in AD models.	MSC‐exosomes improved cognition and brain metabolism in AD models, showing promise as a cell‐free AD therapy.	[332]
Human umbilical cord MSCs cultured on 3D or 2D	miRNAs and proteins	i.v.	3D‐Exo had distinct molecules, boosted α‐secretase, reduced β‐secretase, lowered Aβ, and improved memory.	3D‐Exo from hUC‐MSCs reduced Aβ and improved cognition, promising for AD treatment.	[333]
Adipose‐derived MSCs	miRNA‐22	i.v.	Exo‐miRNA‐22 improved memory and motor function, reduced inflammation and pyroptosis, and boosted neuron survival in APP/PS1 mice.	miRNA‐22 exosomes from ADMSCs helped treat AD by reducing pyroptosis and inflammation.	[313]
MSCs	Functional proteins, mRNA, miRNAs; RVG	i.v.	MSC–RVG‐Exo targeted cortex and hippocampus better, reduced Aβ plaques and astrocyte activation, and balanced inflammatory cytokines (lowered TNF‐α, IL‐1β, IL‐6; increased IL‐4, IL‐10, IL‐13).	Improved memory, lowered amyloid plaques, and reduced inflammation in APP/PS1 mice	[334]

Abbreviations: AD, Alzheimer's disease; ADMSC, adipose‐derived mesenchymal stem cell; ADSC, adipose‐derived stem cell; APP/PS1, amyloid precursor protein/presenilin 1 (transgenic mouse model); Aβ, amyloid beta; Bcl‐2, B‐cell lymphoma 2 (antiapoptotic protein); BDNF, brain‐derived neurotrophic factor; BMSC, bone marrow mesenchymal stem cell; CoQ10, coenzyme Q10; DPSC, dental pulp stem cell; hUC‐MSC, human umbilical cord mesenchymal stem cell; i.n., intranasal; i.p., intraperitoneal; i.v., intravenous; ICAM‐1, intercellular adhesion molecule 1; IL, interleukin; LFA‐1, lymphocyte function‐associated antigen 1; miRNA, microRNA; MSC, mesenchymal stem cell; NEP, neprilysin; NFT, neurofibrillary tangle; RVG, rabies virus glycoprotein; SOX2, SRY (sex determining region Y)‐box transcription factor 2; Tau, microtubule‐associated protein tau; TNF‐α, tumor necrosis factor‐alpha.

**TABLE 5:** Overview of cell‐derived exosomes and their therapeutic applications in PD.

Source of exosomes	Cargo	Administration rout	Key findings	Outcome	References
Blood‐derived exosomes	Dopamine	i.v.	Blood exosomes naturally target the brain and deliver dopamine, increasing brain dopamine levels.	Improved therapeutic efficacy and reduced systemic toxicity	[170]
Macrophage‐derived exosomes	Polydopamine carbon dots	i.v.	PEs cross the BBB, target inflamed brain areas, reduce oxidative stress and neuroinflammation, lower α‐synuclein, and repair neurons.	Significant improvement in both motor and nonmotor symptoms of PD mice	[335]
Bovine milk‐derived exosomes	Epicatechin gallate	In vitro	ECG delivered into SH‐SY5Y cells via exosomes showed better neuroprotection than free ECG by inhibiting apoptosis and mitophagy.	Enhanced neuroprotective effect against rotenone‐induced cell damage	[336]
Immature dendritic cell‐derived exosomes	Cur	i.v.	Engineered exosomes cleared α‐synuclein aggregates and reduced immune activation.	Significantly improved motor behavior and neuroprotection in PD mice	[337]
Exosomes modified with RVG	siRNA targeting alpha‐synuclein (α‐syn)	i.v.	RVG‐exosomes delivered siRNA in the brain, lowering α‐Syn levels and reducing neuron aggregates.	Widespread reduction of α‐Syn aggregates, demonstrating potential to delay or reverse PD pathology	[338]
Modified exosomes expressing RVG	shRNA minicircles targeting alpha‐synuclein	i.v.	RVG‐exosomes delivered shRNA‐MCs to the brain, lowering target protein for 6 weeks, reducing α‐synuclein buildup and neuron loss.	Improved clinical symptoms and demonstrated potential for long‐term treatment	[339]
Exosomes modified with neuron‐targeting RVG peptide	DNA aptamers that specifically bind α‐synuclein	i.p.	Aptamers loaded into RVG‐exosomes reached neurons, reduced α‐synuclein clumps, and protected synapses and neurons.	Reduced pathological α‐synuclein aggregates and improved motor impairments	[340]
Adipose‐derived stem cells	microRNA (miR)‐188‐3p	i.v.	miR‐188‐3p exosomes blocked autophagy and pyroptosis by targeting CDK5 and NLRP3, boosting cell growth.	Potential therapeutic effect on PD by reducing neuronal injury and inflammation, suggesting miR‐188‐3p as a new therapeutic target	[341]
MSCs	Cur	i.n.	PR‐EXO/PP@Cur crosses membranes to deliver drugs, reduces α‐synuclein, and helps neuron recovery and inflammation.	Improved movement and coordination in PD mice; promising treatment for PD and similar diseases	[342]
MSCs	siRNAs targeting m6A demethylase FTO	i.p.	FTO increased and m6A decreased. FTO stabilizes ATM mRNA via m6A. Reducing FTO lowers α‐Syn and protects TH. MSC‐Exo delivers si‐FTO to the brain striatum.	Strongly lowered α‐Syn expression; less dopaminergic neuron death; TH expression restored; Combined effect reduces neuron death by regulating ATM via m6A	[343]
Engineered exosomes with RVG peptide	sgRNA and dCas9‐DNMT3A	i.n.	Targeted SNCA methylation, reduced α‐synuclein, improved motor function, rescued neurons	Alleviated PD progression and neuronal damage through epigenetic regulation	[344]

Abbreviations: ATM, ataxia telangiectasia mutated (protein kinase); BBB, blood–brain barrier; CDK5, cyclin‐dependent kinase 5; Cur, curcumin; dCas9–DNMT3A, catalytically dead CRISPR‐associated protein 9 fused with DNA (cytosine‐5)‐methyltransferase 3 alpha; ECG, epicatechin gallate; Exo, exosome; FTO, fat mass and obesity‐associated protein; i.n. intranasal; i.p. intraperitoneal; i.v. intravenous; m6A, N6‐methyladenosine; MCs, minicircles; miR/miRNA, microRNA; MSC, mesenchymal stem cell; NLRP3, NOD‐, LRR‐, and pyrin domain‐containing protein 3; PD, Parkinson's disease; PEs, polydopamine carbon dot‐loaded exosomes; RVG, rabies virus glycoprotein; sgRNA, single guide RNA; shRNA, short hairpin RNA; siRNA, small Interfering RNA; SNCA, synuclein alpha; TH, tyrosine hydroxylase; α‐Syn, alpha‐synuclein.

**TABLE 6:** Overview of cell‐derived exosomes and their therapeutic applications in other NDs.

Disease model	Source of exosomes	Cargo	Administration rout	Key findings	Outcome	References
HD	Engineered cell line overexpressing miR‐124	miR‐124	i.n.	Downregulation of REST (RE1‐silencing transcription factor) in brain tissue	Proof of concept achieved; no significant behavioral improvement observed	[345]
ALS	Mesenchymal stem cell	miRNAs and antioxidant/anti‐inflammatory gene transcripts	In vitro	Promoted neurite growth, improved morphology, carried protective miRNAs	Demonstrated neuroprotective effects, potential for ALS therapy development	[346]
	Adipose‐derived stem cells	Cell‐derived factors	In vitro	Reduced SOD1 aggregation, normalized phospho‐CREB/CREB ratio and PGC‐1α expression	Improved mitochondrial function and cellular phenotypes in ALS model; potential ALS therapy	[347]
	Adipose‐derived stem cells	Cell‐derived factors	i.n. and i.v.	Improved motor performance, protected motoneurons, NMJ, and muscle; reduced glial activation	Slowed ALS progression; ASC‐exosomes show promise as a therapeutic approach	[348]
	Adipose‐derived stem cells	Not specified	In vitro	Restored complex I activity, improved ETS efficiency, and mitochondrial membrane potential	Rescued mitochondrial dysfunction in ALS cell model; potential therapeutic strategy for ALS	[349]
MS	Mouse neural stem cells	Bryostatin‐1 (Bryo) + PDGFRα ligand	In vitro	Targeted exosomes delivered Bryo to OPCs, boosting remyelination and reducing brain damage and inflammation.	Improved myelin protection and regeneration; potential therapeutic strategy for demyelinating diseases	[350]
	Macrophages	Resveratrol	i.n.	Bio‐orthogonal labeled exosomes target CNS microglia; RSV&Exo reduce inflammation in CNS and body.	Improved clinical symptoms in MS mouse model; demonstrated potential for CNS‐targeted therapy	[351]
	Bone marrow mesenchymal stem cells	Not specified	i.v.	Lowered CNS inflammation and demyelination; changed microglia from M1 to M2; raised anti‐inflammatory cytokines (IL‐10, TGF‐β); cut proinflammatory cytokines (TNF‐α, IL‐12).	Improved neural behavior and reduced CNS damage in EAE rats; potential therapy for autoimmune/inflammatory diseases	[352]

Abbreviations: ALS, amyotrophic lateral sclerosis; ASC, adipose‐derived stem cell; BM‐MSC, bone marrow mesenchymal stem cell; CNS, central nervous system; CREB, cAMP response element‐binding protein; EAE, experimental autoimmune encephalomyelitis; ETS, electron transport system; HD, Huntington's disease; i.n., intranasal; i.v., intravenous; IL, interleukin; M1/M2, microglia activation states; miR / miRNA, microRNA; MS, multiple sclerosis; NMJ, neuromuscular junction; OPC, oligodendrocyte progenitor cell; PDGFRα, platelet‐derived growth factor receptor alpha; PGC‐1α, peroxisome proliferator‐activated receptor gamma coactivator 1‐alpha; REST, RE1‐silencing transcription factor; RSV, resveratrol; SOD1, superoxide dismutase 1; TGF‐β, transforming growth factor beta; TNF‐α, tumor necrosis factor alpha.

### 5.1 Exosome‐Based DDSs for AD

AD is a progressive neurodegenerative condition characterized by its untreatable nature and significant difficulties in clinical management. Cholinesterase inhibitors, such as donepezil, galantamine, and rivastigmine, function as first‐line pharmacological treatments; however, their efficacy is primarily confined to the alleviation of symptoms and fails to address the underlying pathophysiological mechanisms, including the accumulation of amyloid plaques and NFTs [353]. The therapeutic responses to these agents exhibit considerable variability among patients and tend to decline over time, frequently accompanied by adverse side effects [354]. Consequently, AD persists as a formidable challenge within the healthcare domain [355]. Recently, exosomes have garnered attention as promising nanoscale vehicles for drug delivery in therapeutic approaches targeting the brain. A variety of research efforts focus on improving exosome‐based delivery systems by developing specific targeting peptides and employing various drug‐loading techniques, with the goal of optimizing therapeutic outcomes in AD. Table 4 summarizes recent sources and applications of exosomes in AD.

Numerous studies have highlighted the promise of exosome‐based delivery systems for treating AD. Drug‐loaded exosomes represent promising approach. Wang et al. [173] developed a curcumin‐loaded exosome (Exo‐cur) sourced from macrophages, which demonstrated notable neuroprotective effects. Exo‐cur facilitated the targeted delivery of curcumin by binding to the LFA‐1 integrin present on the exosomal surface and ICAM‐1 found on ECs, effectively crossing the BBB. In the brain regions treated, there was a significant increase in curcumin fluorescence intensity, which colocalized with NeuN‐positive neurons in the hippocampus. When compared with the control group treated with okadaic acid (OA), Exo‐cur notably reduced neuronal damage, promoted neuronal survival, lowered the expression levels of Bcl‐2‐associated X protein (Bax) and caspase‐3, and significantly decreased Tau protein phosphorylation. Measurement of GSK‐3β phosphorylation at Ser9 revealed that Exo‐cur inhibited OA‐induced activation of GSK‐3β while increased AKT expression, thereby preventing Tau phosphorylation via the AKT/GSK‐3β signaling pathway and alleviating cognitive impairments. Likewise, silibinin‐loaded exosomes (Exo‐Slb) derived from macrophages showed effectiveness in preventing Aβ aggregation and enhancing cognitive function in AD mouse models [161]. Treatment with Exo‐Slb successfully inhibited astrocyte activation, reduced the release of inflammatory cytokines, and suppressed NF‐κB signaling, which in turn prevented apoptosis in Aβ‐treated SH‐SY5Y cells [161]. Additionally, quercetin‐loaded exosomes from plasma (ExoQue) displayed neuroprotective properties in OA‐induced AD mouse models, primarily by inhibiting CDK5 activity [327]. Exo‐Que decreased the formation of insoluble NFTs and downregulated proteins associated with apoptosis, thereby enhancing antiapoptotic effects [327]. Coenzyme Q10, a dietary supplement known for its antioxidative and anti‐inflammatory properties, significantly improved cognitive function in mouse models of AD induced by streptozotocin when coadministered with exosomes [328]. This improvement was achieved through the upregulation of BDNF and sex‐determining region Y‐box 2 (SOX2) expression levels within the hippocampus [328]. Exosome‐mimetic liposomes have emerged as promising complementary DDSs. Fernandes et al. [356] reported that curcumin‐loaded liposomes reduced oxidative stress by 50% in zebrafish embryos and human neuronal cells, promoting neuroprotection with minimal side effects. Although further validation in adult models is needed, these findings suggest a potential synergy between liposome‐based and exosome‐mediated therapies [356].

Furthermore, exosomes are a key element of stem cell secretomes. Exosomes isolated from dental pulp stem cells (DPSCs) [329], BMSCs [330], and adipose‐derived stem cells (ADSCs) contain enzymes such as enkephalinase and insulin‐degrading enzymes, which possess the capacity to degrade Aβ [357]. Notably, DPSCs secrete high levels of enkephalinase into their extracellular matrix, allowing these exosomes to efficiently degrade Aβ1‐42 and reduce its neurotoxicity in SH‐SY5Y neuroblastoma cells [330]. Administration of BMSC‐derived exosomes into the lateral ventricle yields more favorable outcomes compared with intravenous delivery. This intervention not only enhances AD‐like behavior but also inhibits microglial activation and brain inflammation, reduces amyloid burden, restores neuronal integrity, and elevates BDNF expression levels [331]. Chen et al. [332] demonstrated that exosomes derived from MSCs provide significant therapeutic advantages for AD patients by facilitating Aβ plaque degradation, improving brain glucose metabolism and cognitive abilities, and modulating epigenetic modifications and gene expression. Exosomes extracted from Wharton's jelly MSCs (WJ‐MSC) frequently carry miR‐29a, which directly targets HDAC4, an enzyme found to be elevated in the nuclei of brain tissue of AD patients. Moreover, hippocampal NSC‐derived exosomes carrying miRNAs such as miR‐322, miR‐17, and miR‐485 have demonstrated strong therapeutic potential in preclinical models. These vesicles effectively reduce Aβ oligomer‐induced neurotoxicity, improve memory function, and enhance synaptic plasticity. Together, these effects highlight the promise of exosome‐based strategies for cognitive recovery in AD [358].

The implementation of genetic engineering techniques further enhances the therapeutic potential of exosomes. Jahangard et al. [179] targeted BACE1 through the transfection of miR‐29b into rat BMSCs, resulting in the generation of engineered exosomes. In vitro analyses confirmed the efficient delivery of miR‐29b to target cells via these engineered exosomes. Intra‐hippocampal administration of the engineered exosomes significantly improved learning capabilities in a β‐amyloid‐induced AD animal model. Yang et al. [333] conducted a comparative analysis between exosomes derived from stem cells cultured in 3D systems and those obtained from two‐dimensional (2D) cultures. Their findings revealed that exosomes from human MSCs grown in 3D environments markedly promoted β‐amyloid degradation in APP/PS1 transgenic mouse models, reduced amyloid aggregation, facilitated the clearance of amyloid plaques, and improved both cognitive function and memory. Another study demonstrated that exosomes isolated from MSCs of AD model mice, when loaded with miR‐22 (Exo‐miR‐22), were capable of effectively suppressing pyroptosis by targeting Gasdermin D [359]. This action led to a reduction in the release of proinflammatory mediators and helped to modulate the inflammatory response [359].

Despite their advantageous capacity to traverse the BBB, exosomes predominantly accumulate in the spleen and liver, leading to insufficient brain localization and potential toxicity in nontarget organs [360]. Thus, there is a pressing demand for the creation of advanced exosomes that can accurately target distinct areas of the brain. To improve the specificity and effectiveness of drug delivery, recent research has focused on engineering exosomes by incorporating modifications, such as rabies virus glycoprotein (RVG). The study by Alvarez‐Erviti et al. [313] highlighted the role of exosomes in facilitating the delivery of siRNA. By inhibiting BACE1 expression, they ameliorated the neuropathological conditions observed in AD model mice. They engineered targeted exosomes through the transfection of dendritic cells with a plasmid encoding RVG peptides fused with lysosomal‐associated membrane protein 2b (LAMP2b). The modified exosomes, carrying therapeutic agents such as GAPDH siRNA, were able to cross the BBB after intravenous administration, efficiently delivering their contents to the brain, thereby reducing β‐amyloid production and alleviating neurodegenerative effects linked to AD. Similarly, Cui et al. [334] developed an additional engineered exosome by chemically conjugating RVG to MSC‐derived exosomes (MSC–RVG‐Exo). Due to RVG's specific affinity for acetylcholine receptors, these exosomes were efficiently internalized by neuronal cells. The results demonstrated that RVG‐modified exosomes precisely targeted the hippocampus and cortex, leading to a marked decrease in β‐amyloid plaque accumulation and GFAP expression. This effect was achieved by suppressing astrocyte activation and regulating the inflammatory response in AD models, which in turn significantly improved spatial learning and memory functions. Yu et al. [202] employed a more advanced genetic engineering approach by cotransfecting ADSCs with plasmids encoding RVG, human LAMP2b, and CD10 DM (enkephalinase) fusion proteins to isolate RVG‐carrying engineered exosomes. These exosomes integrated CD10 DM from the transfected cells, selectively binding to human β‐amyloid and facilitating its degradation. In vivo investigations demonstrated that these modified exosomes effectively traversed the BBB and preferentially concentrated in the hippocampus, fostering an anti‐inflammatory environment conducive to the treatment of AD. This therapeutic benefit was achieved by increasing the levels of anti‐inflammatory cytokines, such as IL‐10, coupled with a reduction in proinflammatory cytokines, including IL‐1α, TNF‐α, and NF‐κB.

Exosome‐based therapies are rapidly evolving to enhance brain‐specific delivery and reduce off‐target effects. Advanced techniques such as peptide conjugation and CRISPR–Cas9 gene editing are expanding therapeutic precision. Notably, exosome‐mediated CRISPR–Cas9 delivery has shown promise in correcting ApoE4‐related mutations in AD. The engineered exosome platform MAPLEX utilizes mMaple3 for protein loading and blue‐light‐triggered release, enabling targeted epigenetic editing. In AD mouse models (5xFAD, 3xTg‐AD), MAPLEX‐delivered sgRNA against Bace1 reduced amyloid plaques and improved cognition. These findings highlight exosomes as promising nanocarriers for ND treatment.

Combining exosomes with treatments like aducanumab can improve drug delivery across the BBB and enable sustained release. Additionally, delivering exosomes with neurotrophic and anti‐inflammatory factors may restore synapses, reduce inflammation, and boost cognition, expanding therapies for AD.

### 5.2 Exosome‐Based DDSs for PD

PD, the most common synucleinopathy [361], is predominantly treated using a combination of pharmacological therapies, surgical interventions, and rehabilitative strategies. Medications like levodopa and dopamine agonists provide symptomatic relief; however, they are frequently accompanied by undesirable side effects [362]. Deep brain stimulation, entailing the implantation of electrodes into targeted brain regions, serves as an additional therapeutic strategy for managing symptoms. Moreover, physical and speech therapies play a crucial role in enhancing motor skills and communication abilities. Nonetheless, no definitive cure for PD has been established, which continues to fuel intensive research aimed at discovering more effective therapeutic approaches. Table 5 summarizes recent sources and applications of exosomes in PD.

Haney et al. [363] pioneered an exosome‐mediated therapeutic approach for PD by encapsulating the antioxidant enzyme catalase in exosomes. In vitro studies demonstrated that catalase‐loaded exosomes exhibited a preferential accumulation in neurons and microglial cells. Following IN delivery in a PD mouse model, notable levels of these exosomes were detected within the brain, highlighting their neuroprotective effects. Additionally, an independent investigation showed that exosomes derived from PD patients, when introduced into wild‐type mice, induced significant molecular changes and impaired motor functions [364]. Expanding on these findings, Kojima et al. [365] engineered an exosome formulation encapsulating catalase mRNA, which, upon intravenous administration, specifically targeted the brains of 6‐OHDA‐induced PD mouse models, leading to significant reductions in neurotoxicity and neuroinflammation. Ren et al. [340] developed RVG‐exosomes carrying aptamer F5R2 to specifically target α‐syn fibrils. These exosomes reduced α‐syn aggregation in the brain, improved motor function, and spared normal α‐syn, offering a potential treatment for synucleinopathies [340].

A key obstacle in the treatment of PD is the effective delivery of dopamine to the brain, a process that is significantly hindered by the BBB. Although dopamine encapsulation in nanoparticles improves BBB permeability, several limitations remain unresolved. In 2018, Qu et al. [170] devised a novel approach utilizing supersaturated solution incubation to encapsulate dopamine within blood exosomes. These exosomes, which are able to traverse the BBB through interactions with TFRs, resulted in a 15‐fold enhancement in dopamine distribution within the brain. When administered intravenously to mouse models of PD, dopamine‐encapsulated exosomes exhibited lower toxicity and superior therapeutic efficacy compared with free dopamine. This treatment promoted dopaminergic neurogenesis, elevated endogenous dopamine concentrations, and led to improved neurobehavioral performance. Moreover, Yang et al. [335] developed a novel nano‐delivery system composed of polydopamine carbon dots (PDA C‐dots) encapsulated within macrophage‐derived exosomes (PEs). The PDA C‐dots demonstrated both antioxidant and anti‐inflammatory activities, while the exosomal carrier markedly improved BBB penetration and targeted delivery to inflammatory sites via LFA‐1/ICAM‐1‐mediated intrinsic tropism [335].

Despite its recognized neuroprotective properties, epigallocatechin gallate (EGCG) encounters significant obstacles related to stability and bioavailability [366]. Exosomes represent a promising delivery platform. For instance, EGCG‐loaded exosomes (EGCG‐Exo) have been shown to inhibit mitophagy via the PINK1/parkin pathway, thereby safeguarding SHSY5Y cells and minimizing apoptosis [336]. Sharma demonstrated that Exo‐Cur outperformed siRNA‐loaded exosomes in reducing ROS levels, preventing α‐syn aggregation, and mitigating neurotoxicity [367]. Recent progress has resulted in the creation of hybrid nanoparticles, comprising a gene‐chemical core enclosed within a shell modified by RVG‐exosomes. These nanoparticles function as nano‐scavengers, effectively reducing α‐syn aggregation and enhancing motor function in PD models [337].

A key therapeutic approach in the treatment of PD continues to be the targeting of α‐syn fibril aggregation [368]. One promising approach involves leveraging modified exosomes to deliver therapeutic agents targeting pathogenic α‐syn aggregates, a hallmark of PD pathology. Cooper et al. [338] demonstrated that modified exosomes effectively delivered siRNA targeting α‐syn, leading to a significant reduction in both total and aggregated α‐syn levels in the brains of PD mouse models. Additionally, systemic administration of these siRNA‐loaded exosomes, utilizing RVG‐expressing exosomes, further diminished α‐syn mRNA and protein levels in dopaminergic neurons of the substantia nigra. Nevertheless, the short‐term effects of siRNA therapy pose challenges to its long‐term therapeutic effectiveness. Izco et al. [339] suggested that shRNA delivered via RVG‐exosomes could offer a more sustained therapeutic benefit by persistently targeting and reducing α‐syn aggregation. In another study, Ren et al. [340] showed that aptamers delivered via RVG‐exosomes effectively diminished pathogenic preformed fibrils and mitigated motor deficits in PD models. The aptamer F5R1, a selective α‐syn‐binding molecule, successfully inhibited α‐syn aggregation. This inhibition was accompanied by an alleviation of cellular and mitochondrial dysfunction. In PD mouse models, these modified exosomes substantially reduced α‐syn aggregation and restored motor function. Yang et al. [171, 369] used MSC‐derived exosomes to deliver ASOs into the brains of A53T mutant transgenic PD mice. This approach effectively reduced α‐syn expression and showed promising potential to alleviate disease symptoms.

Li et al. [341] demonstrated that exosomes can function as efficient vectors for gene therapies aimed at specific brain regions, leading to improvements in motor performance and memory in mice. Furthermore, the potential of exosomal miRNAs as therapeutic tools for PD has been investigated. Research indicates that exosomes carrying miR‐188‐3p inhibit autophagy and pyroptosis, while those loaded with miR‐30a‐5p alleviate motor dysfunction and lessen pathological changes characteristic of PD [341, 370]. Peng et al. [342] designed a self‐targeting nanosystem (PR‐EXO/PP@Cur), in which exosomes, modified with penetratin and RVG peptides, were loaded with miR‐133b, curcumin, and superparamagnetic iron oxide nanoparticles. Serum‐derived exosomes loaded with miR‐137 significantly reduce oxidative stress in neurons, leading to improved physiological function and behavioral performance in PD animal models [371]. Additionally, MSC‐Exo carrying siRNA targeting the m6A demethylase FTO (si‐FTO) substantially reduced α‐syn expression and limited dopaminergic neuron death in PD mouse models [343].

Exosome delivery of CRISPR/Cas9 provides a new way to target genetic causes of PD. Kong et al. [344] created engineered exosomes (RVG‐CRISPRi‐Exo) guided by FUS to deliver CRISPR to brain lesions, causing targeted methylation of the SNCA gene. This reduced α‐syn levels, improved motor function, and protected neurons in PD models. The study highlights the potential of precise exosome‐based gene editing for treating NDs [344].

A recent study by Huang et al. [372] demonstrated that intranasally administered exosomes derived from umbilical cord MSCs can cross the BBB and be internalized by neurons and glial cells in a PD model. This intervention led to significant improvements in both motor and nonmotor symptoms, preserved dopaminergic neurons in the substantia nigra, increased neuronal activity in the olfactory bulb, and attenuated neuroinflammation by suppressing microglial and astrocyte activation. These findings highlight the therapeutic promise of exosome‐based nanomedicine for PD [372].

### 5.3 Exosome‐Based DDSs for HD

HD is uniquely characterized among neurodegenerative disorders by its specific genetic target, the *HTT* gene. This specificity renders gene‐targeted therapies a highly promising avenue of research, despite the fact that such strategies do not consistently target the *HTT* gene directly [373].

Among the diverse therapeutic strategies available, miRNA‐based interventions have shown substantial potential for the treatment of NDs [374]. Notably, MiR‐124 is frequently employed in exosome‐based therapies, given its elevated expression in nearly all brain regions, apart from the pituitary gland, where its expression is over 100‐fold lower. This miRNA plays a pivotal role in regulating both CNS development and pathology [375]. Additionally, miR‐124 promotes adult neurogenesis and boosts BDNF expression by inhibiting the repressor RE1‐silencing transcription factor (REST) [376]. Notably, miR‐124 is one of the most significantly downregulated miRNAs in HD. Research has shown that miR‐124 can slow the progression of HD in R6/2 transgenic mice by facilitating neuronal differentiation and enhancing neuronal survival [377]. In 2017, Lee et al. [345] developed an exosome‐based platform for delivering miR‐124 by engineering HEK293 cells to produce exosome‐rich miR‐124.

When injected into the striatum of R6/2 HD mice, these exosomes led to a significant decrease in REST protein levels. Nevertheless, no notable improvement in Rotarod performance was detected within 1 week postadministration, and the potential for long‐term effects remains to be comprehensively assessed. In light of these modest results, Lee et al. [345] suggested increasing the dosage of miRNA in exosomes and recommended exploring additional miRNAs, including miR‐9, miR‐22, miR‐125b, miR‐146a, miR‐150, and miR‐214, which may offer improved therapeutic efficacy.

siRNAs function by binding to target mRNAs and facilitating their degradation through the RNA‐induced silencing complex, thus enabling effective and sustained gene silencing [378, 379]. Wu et al. [380] examined the therapeutic effectiveness of siRNA delivery via exosomes in BACHD and N171‐82Q mouse models of HD.

Modified exosomes, expressing neuron‐specific RVG peptides and carrying siRNAs targeting human huntingtin exon 1 (HuHTT) transcripts, were utilized in this study. These RVG‐tagged exosomes containing HuHTT–siRNA were administered intravenously to normal and HD mice at a dose of 10 mg/kg every other day for a duration of 2 weeks. This treatment effectively delivered siRNA to the brain, leading to a significant reduction in HTT expression, with decreases of 46% in BACHD mice and 54% in N171‐82Q mice. Moreover, the N171‐82Q mice showed enhanced motor performance in the Rotarod test. This research underscores the potential of HuHTT–siRNA‐loaded RVG exosomes as a viable therapeutic approach for HD. In a complementary study, Zhang et al. [172] integrated natural exosomal systems with engineered genetic circuits to develop a system driven by a cytomegalovirus promoter. This system encodes RVG‐targeted peptides along with mHTT‐silencing siRNAs. Upon intravenous administration, RVG‐labeled exosomes loaded with mHTT siRNA were produced and effectively reached the cortex and striatum. This method led to a decrease in mHTT protein levels, a reduction in toxic aggregate formation, enhanced behavioral outcome, and lessened neuropathological damage in the striatum and cortex of mice with HD.

Hydrophobically modified siRNA (hsiRNA) is a chemically engineered oligonucleotide designed to improve stability and facilitate cellular uptake. These siRNAs effectively bind to cell membranes, enter cells, and initiate gene silencing. While exosomes present a promising natural nanocarrier for RNA delivery, challenges persist in achieving sufficient loading of RNA quantities [381]. In response to this challenge, Bicans et al. [381] developed a series of cholesterol‐conjugated hsiRNAs, which were subsequently incorporated into exosomes. In comparison with free cholesterol–hsiRNA, exosome‐loaded cholesterol–hsiRNA demonstrated greater efficacy in silencing *HTT* mRNA in neurons. However, cholesterol–hsiRNA delivered through larger EVs did not achieve significant target mRNA silencing. In a different study, hsiRNAs targeting huntingtin RNA were incorporated into exosomes via a simple coincubation method [379]. In vitro experiments revealed that primary cortical neurons efficiently internalized exosome‐encapsulated hsiRNAs, which resulted in a dose‐dependent decrease in both *HTT* mRNA and protein levels. Furthermore, when hsiRNA‐loaded exosomes were infused unilaterally into the striatum of mice, they exhibited bilateral distribution throughout both the striatum and cortex, leading to a significant reduction in *HTT* mRNA expression. In contrast, free hsiRNA lacking exosomal encapsulation did not produce comparable effects, thereby reinforcing the viability of exosomes as effective delivery systems for therapeutic oligonucleotides in the treatment of NDs [379].

Exosomes can deliver CRISPR–Cas9 to precisely edit the mHTT gene, enabling permanent genome modification that selectively targets the harmful allele while sparing the normal one, thus minimizing off‐target effects. Beyond gene editing, exosomes can also transport molecules that enhance autophagy to clear toxic mHTT aggregates. Codelivering siRNA and autophagy activators via exosomes may synergistically improve mHTT clearance, promote neuron survival, and reduce HD symptoms.

### 5.4 Exosome‐Based DDSs for ALS

The treatment of ALS poses considerable challenges. Despite the comprehensive evaluation of various pharmacological agents in clinical trials, the majority of therapies achieve only a modest attenuation of disease progression. The agents include repurposed drugs originally approved for cancer treatment (such as bosutinib and masitinib), antiretroviral therapies for HIV (including dolutegravir, abacavir, and lamivudine), and medications for rheumatoid arthritis (like baricitinib). Additionally, there are preclinical candidates comprising autophagy inducers (e.g., rapamycin), hormone antagonists (such as tamoxifen and imatinib), alkylating agents (including cisplatin and carboplatin), and immunomodulatory agents (e.g., thalidomide and lenalidomide) [382, 383, 384, 385, 386]. Nonpharmacological interventions, such as respiratory support, nutritional management, and physical therapy, yield transient benefits but demonstrate limited efficacy during the advanced stages of the disease.

In this framework, exosomes derived from MSCs exhibit strong neuroprotective properties by delivering functional cargos such as miRNAs, proteins, and lipids to neural cells. Notably, Gschwendtberger et al. [346] reported that the exosomal transfer of neurotrophic factors markedly attenuated neurotoxicity in ALS‐associated motor neurons.

Exosomes derived from ADSCs (ADSC‐exo) have shown significant therapeutic benefits in preclinical ALS models. Studies indicate that ADSC‐exo can reduce *SOD1* aggregation in the G93A ALS mouse model, lower cytosolic *SOD1* levels, and restore the p‐CREB/CREB ratio as well as PGC‐1α expression [347]. These results imply that ADSC‐exo can influence cellular phenotypes linked to ALS, particularly those associated with SOD1 aggregation and mitochondrial dysfunction [347]. Additionally, repeated administration of ADSC‐exo has been shown to effectively target affected areas, improve motor function, and decrease glial activation [348]. Calabria et al. [349] further emphasized the therapeutic efficacy of ADSC‐derived exosomes in reinstating mitochondrial complex I activity, membrane potential, and the performance of the electron transport chain. Wang et al. [387] found that hADSC‐derived exosomes reduced motor neuron damage by lowering oxidative stress and inflammation. Schwann cell‐derived exosomes also showed potential, with one ALS patient experiencing symptom relief through the United States Food and Drug Administration (US FDA)’s Expanded Access Program [388]. Mazzini et al. [389] reviewed stem cell‐based approaches and highlighted exosomes as key mediators in translating preclinical results to clinical trials. Recently, the US FDA approved clinical testing of Aruna Bio's exosome therapy AB126 for ALS, marking a step toward clinical use [390].

To improve the targeting of exosomes to regions affected by ALS, researchers have employed genetic engineering techniques to modify the surfaces of exosomes with specific targeting peptides, including the RVG peptide. Alvarez‐Erviti et al. [313] adopted this approach by expressing RVG on the exosomal surfaces, thus enabling the targeted delivery of siRNA to the brain and significantly reducing the expression of target gene. Although this research primarily focused on AD, the underlying principles and methods may also be relevant to ALS. Additionally, Kojima et al. [365] showed that genetically engineered exosomes delivering miRNA could improve disease phenotypes in ALS mouse models, thereby enhancing therapeutic delivery efficiency and modestly slowing disease progression.

### 5.5 Exosome‐Based DDSs for MS

Immunosuppression presently constitutes a central objective in the therapeutic management of MS to mitigate demyelination, a process potentially arising from several deleterious immunological responses [391]. Although therapeutic approaches for progressive MS remain ambiguous and multiple pharmacological agents show promise in facilitating remyelination, none has thus far exhibited effectiveness in repairing damaged myelin [392]. Consequently, there is a pressing demand for therapies that specifically focus on remyelination. The application of exosome‐mediated delivery systems for mRNA and various therapeutic agents aimed at enhancing remyelination and facilitating neural recovery in MS presents considerable potential for therapeutic progress.

Investigations into EVs derived from various cellular sources have produced encouraging results. Exosome‐based therapies have demonstrated the capacity to regulate immune responses by downregulating proinflammatory cytokine production while enhancing regulatory T (Treg) cell activity. Ojeda‐Hernández et al. [393] emphasized the effectiveness of engineered exosomes in attenuating neuroinflammatory processes and facilitating remyelination.

For example, Wu et al. [350] extracted exosomes from NSCs in murine models and modified them with lentivirus‐armed PDGFR ligands to create targeted exosomes intended specifically for the delivery of mossy fibronectin‐1. This novel approach not only provided enhanced protection for myelin but also facilitated remyelination, accompanied by suppression of astrocyte proliferation, axonal injury, and the activation of proinflammatory microglia. Microglia play a crucial role in monitoring the CNS, and a disruption in their M1/M2 phenotypic balance has been linked to the pathogenesis of MS [394]. EVs derived from dendritic cells that overexpress TGF‐β1 have been shown to inhibit the differentiation of Th1 and Th17 cells, promote the development of Treg cells, and thereby result in a milder EAE phenotype [395]. Furthermore, researchers have employed macrophage‐derived exosomes as natural carriers for delivering resveratrol to the CNS, effectively reducing inflammation in both central and peripheral nervous systems [351]. The progression of MS is associated with the activation of various cytokines, such as TNF‐α and IL‐12, which are critical in triggering inflammation and causing damage to the myelin sheath. Conversely, IL‐10 and TGF‐β are considered promising therapeutic targets for managing this condition [396]. Exosomes secreted by BMSCs have shown the ability to enhance neurobehavioral outcomes, to diminish inflammatory cell infiltration in the CNS, and to reduce demyelination. This study also indicated increased levels of M2‐associated cytokines (IL‐10 and TGF‐β) and decreased levels of M1‐associated cytokines (TNF‐α and IL‐12) following exosome administration [352]. Additionally, intravenous delivery of exosomes derived from IFN‐γ‐stimulated MSCs resulted in decreased demyelination and neuroinflammation, accompanied by the proliferation of Treg cell in the spinal cords of EAE mice [397].

In the EAE mouse model of MS, administering exosomes derived from the serum of pregnant mice or human periodontal membrane stem cells resulted in significant improvements, likely due to the suppression of the Th1 immune response.

Additionally, exosomes obtained from glioblastoma cells that were loaded with curcumin resulted in a delay and reduction of clinical symptoms in mice [398]. This effect is potentially mediated by mechanisms related to immune tolerance and the apoptosis of activated immune cells, which indicates that exosomes could serve as effective vehicles for delivering anti‐inflammatory medications [399]. MSCs have consistently shown enhanced effectiveness in the treatment of EAE and are currently undergoing assessment in clinical trials for MS [400]. MSCs derived from placental tissues exhibit regenerative, protective, and immunomodulatory characteristics. Research suggests that treatment with exosomes from these cells decreases DNA damage in the spinal cord, accompanied by enhanced myelination [401]. In a similar vein, the systemic delivery of exosomes originating from bone marrow‐derived MSCs has been shown to diminish immune cell infiltration and inflammation within the CNS, alleviate demyelination, and promote enhanced myelination [402]. Studies have indicated that bone marrow‐derived MSCs that have been pretreated with IFN‐γ can improve the condition of EAE by inhibiting the activation of pathogenic T cells and boosting the activity of regulatory Treg cells in animal models [397]. These results highlight the significant potential of therapies based on exosomes for addressing autoimmune and CNS disorders.

In an in vitro model of MS triggered by lysophosphatidylcholine, exosomes extracted from the serum EVs of young rats, enriched with miR‐219, significantly reduced oxidative stress and simultaneously enhanced the levels of oligodendrocyte precursor cells and myelin content within the hippocampus [400]. Furthermore, the intravenous administration of these EVs decreased brain atrophy, promoted the proliferation of NSCs in the subventricular zone, and lowered the serum levels of inflammatory cytokines in mice infected with Theiler's murine encephalomyelitis virus, which is utilized as a model for progressive MS. EVs facilitated the reduction of motor deficits and enhancement of passive avoidance memory (i.e., the ability to avoid noninvasive foot shocks) in infected animals by promoting remyelination and alleviating brain atrophy [403]. In the experimental models of MS induced by EAE and cuprizone diet, exosomes derived from MSCs efficiently traversed the BBB. This led to a significant rise in the quantity of newly formed neurons, suppression of the TLR2/IRAK1/NFκB signaling pathway, and improvement in social behavior assessments. Notably, animals in the cuprizone model exhibited increased time spent in areas with unfamiliar conspecifics following the administration of MSC exosomes [404].

Aptamers, which are short RNA or DNA sequences distinguished by their secondary or tertiary structures that enable selective binding to intracellular proteins or various targets, have attracted significant interest [405]. The LJM‐3064 aptamer has been thoroughly examined due to its high affinity for myelin, serving dual roles as both a targeting ligand and a therapeutic agent, as highlighted in earlier studies [406]. An in vivo investigation revealed that the administration of exosome‐conjugated LJM‐3064 aptamers before exposure to a particular pathogen effectively inhibited the Th1 immune response and increased the population of regulatory Treg cell [407]. The specific biodistribution of exosomes warrants additional research to clarify their in vivo dynamics. Exosomes engineered with RVG peptides have shown a remarkable ability to target the brain, potentially improving drug delivery and therapeutic effectiveness for CNS disorders [408]. To further facilitate the delivery of BDNF to the brain, Zhai et al. [409] created exosomes loaded with BDNF mRNA and modified with RVG peptides. These engineered exosomes, administered intranasally to mice subjected to cuprizone treatment, exhibited significant potential for effective BDNF delivery, promotion of remyelination, and improvement of motor coordination [409].

## 6 Challenges and Limitations of Exosome‐Based Brain‐Targeted DDSs for NDs

The treatment of NDs is critically impeded by the BBB, underscoring the necessity for the development of therapeutic strategies capable of effectively overcoming these formidable challenges. Exosomes are nano‐sized vesicles with a lipid bilayer and released by a variety of cell types into different biological fluids. Their unique characteristics position them as highly promising vehicles for drug delivery in NDs. Numerous investigations have examined the therapeutic efficacy of exosomes in treating neurodegenerative disorders, including AD, PD, HD, ALS, and MS. These investigations demonstrate that exosomes have the capacity to mitigate disease symptoms by fostering neurogenesis, attenuating neuroinflammation, enhancing angiogenesis, and facilitating improvements in synaptic plasticity. Recent progress in exosome research has driven the commencement of multiple promising clinical trials, which are comprehensively summarized in Table 7. For instance, the clinical trial (NCT06082713) aims to identify blood‐based biomarkers for HD progression using EV technology. Concurrently, evaluate the impact of a prolonged, combined aerobic exercise and cognitive training regimen on cognitive performance and blood‐derived exosomal synaptic protein concentrations in elderly individuals at elevated risk for AD (NCT05163626). Notwithstanding these promising features, the clinical utilization of exosomes faces several notable challenges: (1) the absence of standardized isolation and purification protocols; (2) suboptimal loading efficiency of therapeutic agents; (3) insufficient targeting precision; (4) inadequate safety and immunogenicity evaluations; and (5) challenges in clinical translation [410, 411, 412].

**TABLE 7:** Ongoing clinical research on exosomes.

Research period	Related disease	Possible exosome effects	Research purpose	Study type	Estimated enrollment	NCT number
2017/12/20–2023/12	AD	Biomarker	Explore the presence of Tau in EVs in CSF	Observational	100 participants	NCT03381482
2024/12–2034/12	AD	Prevention	Investigate the effect of a long‐term combined aerobic exercise and cognitive training program on cognitive function and blood exosomal synaptic protein levels for seniors at increased risk for AD	Interventional	200 participants	NCT05163626
2013/01–2016/06/21	PD	Biomarker	Determine whether there are biomarkers associated with PD susceptibility and/or progression in exosome‐proteomes; Determine if leucine‐rich repeat kinase 2 (LRRK2) expression and/or phosphorylation are significantly lowered in the exosomes of individuals treated with the potent LRRK2 kinase inhibitor sunitinib (a multikinase inhibitor compound), to establish an assay for on‐target effects for future LRRK2 inhibitor clinical trials	Observational	601 participants	NCT01860118
2022/07/06–2023/12/31	PD	Biomarker	(1) Compare the effects between experimental treatment and conventional treatment; (2) explore whether it is possible to identify predictive and indicative biomarkers of an outcome measure of rehabilitation using EVs	Interventional (clinical trial)	60 participants	NCT05902065
2023/10/25–2031/11	HD	Biomarker	Use EVs to identify a less invasive blood‐based biomarker of brain Huntingtin	Observational	100 participants	NCT06082713

Data sources—Clinical Registration Website (https://clinicaltrials.gov/).

Abbreviations: AD, Alzheimer's disease; CSF, cerebrospinal fluid; EVs, extracellular vesicles; HD, Huntington's disease; LRRK2, leucine‐rich repeat kinase 2; PD, Parkinson's disease.

A primary challenge in the field of exosome research is the absence of standardized techniques for the isolation and purification of exosomes [413]. These vesicles are widely found in various biological fluids, such as blood, saliva, and urine, where they establish close interactions with other biomolecules. Additionally, exosomes exhibit substantial overlap in terms of size and surface characteristics, and their lack of specific subtype markers further exacerbates the challenges in purification [414]. Although numerous isolation methods have been established, differential ultracentrifugation continues to be the most commonly used approach owing to its comparative ease of use. Nevertheless, the high shear forces produced during ultracentrifugation pose risks to exosome integrity, potentially leading to rupture and aggregation. Furthermore, this technique is labor‐intensive and highly dependent on the operator, leading to significant batch‐to‐batch variability, which restricts its broader clinical applicability [415]. Density gradient methods, which exploit density variations between vesicles and protein aggregates, are similarly prone to causing exosome damage due to shear forces and are also complex and time intensive [416]. Immunoaffinity‐based isolation approaches, which rely on specific surface marker proteins to achieve high selectivity, face limitations stemming from the absence of universally recognized exosome markers. Moreover, issues surrounding scalability and clinical applicability remain critical challenges in this domain [417]. Size‐based isolation techniques, such as ultrafiltration, show promise for automation; however, large‐scale production of highly purified exosomes, with preservation of their integrity and biological function, continues to pose a substantial challenge [418]. As a result, the isolation and purification of exosomes remain fraught with complex challenges, necessitating the resolution of numerous biological and technical barriers. There is an urgent need within the scientific community for standardized, efficient isolation techniques to scale up exosome production, thereby accelerating both research and clinical applications. This demand calls for innovative optimization of current techniques and the development of novel separation strategies, informed by a more profound understanding of small EV (sEV) biology, to drive future progress in exosome research.

A principal challenge in exosome‐based drug delivery lies in the limited loading efficiency, which stems largely from the unique structural properties of exosomes [419], Although their phospholipid bilayer shields them from degradation, they simultaneously restricts the available space for the incorporation of exogenous drugs, particularly hydrophilic compounds like RNA. In response to this limitation, researchers have devised various strategies to enhance drug loading. Preloading strategies, such as transfection and coincubation, aim to encapsulate therapeutic agents such as RNA during the exosome formation process within parent cells [420]. However, the efficiency of incorporation largely depends on the type and sequence of the RNA. In contrast, postloading techniques such as incubation, electroporation, sonication, extrusion, and freeze–thaw cycles offer a range of methodologies and generally exhibit improved success rates, particularly for hydrophobic compounds like curcumin [421, 422]. Yet, the lipid bilayer persists as a substantial barrier for hydrophilic compounds such as RNA, significantly curbing passive loading efficiency. To circumvent these challenges, researchers have pursued innovative approaches, including the engineering of parent cells and the synthesis of biomimetic LNPs via microfluidic techniques, aimed at optimizing encapsulation efficiency. Notwithstanding these advancements, attaining high drug‐loading efficiency without compromising the integrity of the exosomal membrane continues to be a significant challenge. Any compromise in membrane integrity risks impairing exosome stability and bioactivity, consequently diminishing their therapeutic efficacy [37, 423]. Therefore, meticulous attention is required to preserve exosome membrane integrity during drug loading to retain their immune‐privileged properties. Furthermore, careful monitoring of the biological properties of exosomes, both pre‐ and postloading, including their specificity and targeting capabilities, is crucial to ensuring that the final therapeutic product complies with safety and efficacy standards.

Exosomes present distinct benefits as DDSs, particularly their capacity to target and accumulate in specific tissues, surpassing traditional delivery vehicles. Nonetheless, significant challenges complicate the realization of precise and targeted delivery. A significant limitation is their nonspecific biodistribution, often resulting in unintended accumulation in nontarget organs such as the liver, lungs, spleen, kidneys, and pancreas. This phenomenon reduces targeting efficiency and increases the likelihood of adverse effects [190]. Nevertheless, unmodified exosomes from certain cell types show relatively high specificity in tissue accumulation, indicating potential for optimization through the strategic selection of parent cells. The creation of hybrid exosomes and the modification of therapeutic cargos demand thorough assessment, as potential adverse effects remain a significant concern. Consequently, rigorous clinical evaluations of both efficacy and safety are essential. The intrinsic complexity of exosome composition, particularly their reliance on donor cells, complicates the detailed characterization of therapeutic cargos and exosome mimetics. Potential immunogenic responses, ranging from immune activation to suppression, introduce further uncertainty into their therapeutic use. To enhance targeting accuracy, ongoing research is concentrating on developing engineering strategies, such as the optimized design of surface proteins aimed at improving binding specificity to particular tissues and cells. This strategy has shown promising results in delivering neuroprotective agents, with increased drug concentration within target cells and reduced associated side effects [424]. Additionally, advances in exosome imaging technologies have provided more precise tools for monitoring exosome dynamics under both physiological and pathological conditions. These innovations enhance our understanding of exosome mechanisms and establish a solid foundation for clinical translation. Nevertheless, ongoing efforts are essential to tackle enduring challenges and broaden the possible applications of exosomes in drug delivery.

From a safety standpoint, sEVs encounter challenges in effectively crossing the BBB. The majority of sEVs fail to adequately penetrate the BBB, leading to systemic accumulation and potential adverse effects, including oxidative stress, apoptosis, and inflammatory responses, all of which pose significant health risks [425]. Moreover, extended retention of sEVs in the body may lead to unanticipated biological effects, particularly when their biodegradability is insufficient. The immunogenicity of sEVs remains difficult to predict. The development of a protein corona on the surfaces of sEVs can hinder the attachment of targeting molecules, thereby diminishing both targeting precision and therapeutic effectiveness. Furthermore, this protein corona could be identified as a foreign antigen by the immune system, triggering immune rejection. These immunogenic factors hinder the reuse of sEVs and amplify uncertainty in their therapeutic applications [426].

Therefore, despite its promise, sEV technology remains in the early stages of clinical translation and faces significant challenges. While basic research has uncovered various biological functions of sEVs, their exact in vivo mechanisms, dose–response relationships, and long‐term safety still require extensive investigation. The translation of sEVs from laboratory research to clinical applications requires overcoming key technical challenges, such as scalability, throughput, and clinical sample screening [97]. Furthermore, sEV dosages must be meticulously adjusted to account for individual patient variations, ensuring both therapeutic efficacy and safety [427].

## 7 Conclusion and Future Perspectives

Exosomes represent a highly advantageous nanoplatform for drug delivery, especially in addressing chronic NDs. They play a crucial role in promoting intercellular communication among brain cells and facilitating the transfer of nucleic acids and therapeutic agents, with low immunogenicity and excellent biocompatibility. Furthermore, exosomes have the capability to modify the surrounding microenvironment, act as biomarkers, prevent the spread of disease‐related molecules, and effectively penetrate the BBB. Bioengineered exosomes have demonstrated notable effectiveness in early clinical trials, particularly in enhancing the targeted delivery of therapeutic agents to brain cells. However, various challenges remain. Insufficient knowledge regarding the biogenesis of exosomes, the mechanisms that govern drug sorting within recipient cells, and their roles in the pathogenesis of NDs continues to impede advancement. Moreover, the impact of glial cell‐derived exosomes on neurogenesis, the heterogeneity observed among isolated vesicles, and the long‐term ramifications of exosome‐based therapies remain poorly understood.

Ongoing investigations are crucial for clarifying the pathways associated with exosome biogenesis and understanding the mechanisms of drug sorting within recipient cells. This knowledge is vital for optimizing exosomes as efficient vehicles for drug delivery. A comprehensive understanding of how brain‐derived exosomes facilitate intercellular communication and drug transport, combined with detailed insights into their biodistribution and pharmacokinetics, is critical. Moreover, it is crucial to implement stringent criteria for the quality and purity of exosomes, along with standardizing key processes such as source selection, isolation, characterization, drug loading, stability, and targeting capabilities. Future investigations should focus on elucidating the precise composition of exosomal cargo and its regulatory mechanisms, with a strong focus on enhancing targeted delivery to mitigate adverse effects. Collaborative interdisciplinary efforts will be crucial in tackling these challenges.

The optimization of exosome production and the enhancement of their specificity for the treatment of NDs remain key objectives. Ongoing research into exosome engineering technologies, along with advancements in imaging techniques for tracking drug delivery, is crucial. Additionally, identifying mechanisms that facilitate BBB penetration is equally important. Furthermore, establishing rigorous criteria for the quality and purity of exosomes is essential to optimizing their therapeutic applications. In conclusion, exosomes represent a novel and promising pathway for the diagnosis and treatment of chronic NDs. However, further research is needed to overcome current limitations and fully realize their clinical potential.

## Author Contributions

M.S. organized the team and revised the manuscript. M.S., F.Q., Q.B., Y.Z., X.Y., D.Z., and X.C. reviewed, edited the manuscript, and participated in the discussion. All authors have read and approved the final manuscript.

## Ethics Statement

No ethical approval was needed.

## Conflicts of Interest

The authors declare no conflicts of interest.

## Data Availability

No additional data are included.
